# *p*-Type Two-Dimensional Semiconductors: From Materials Preparation to Electronic Applications

**DOI:** 10.1007/s40820-023-01211-5

**Published:** 2023-10-18

**Authors:** Lei Tang, Jingyun Zou

**Affiliations:** 1 Songshan Lake Materials Laboratory, Dongguan, 523808 Guangdong People’s Republic of China; 2 Jiangsu Key Laboratory of Micro and Nano Heat Fluid Flow Technology and Energy Application, School of Physical Science and Technology, Suzhou University of Science and Technology, Suzhou, 215009 Jiangsu People’s Republic of China

**Keywords:** Two-dimensional materials, *p*-type semiconductor, Top–down, Bottom–up, Electronics, Optoelectronics

## Abstract

Two-dimensional (2D) materials are regarded as promising candidates in many applications, including electronics and optoelectronics, because of their superior properties, including atomic-level thickness, tunable bandgaps, large specific surface area, and high carrier mobility. In order to bring 2D materials from the laboratory to industrialized applications, materials preparation is the first prerequisite. Compared to the *n*-type analogs, the family of *p*-type 2D semiconductors is relatively small, which limits the broad integration of 2D semiconductors in practical applications such as complementary logic circuits. So far, many efforts have been made in the preparation of *p*-type 2D semiconductors. In this review, we overview recent progresses achieved in the preparation of *p*-type 2D semiconductors and highlight some promising methods to realize their controllable preparation by following both the top–down and bottom–up strategies. Then, we summarize some significant application of *p*-type 2D semiconductors in electronic and optoelectronic devices and their superiorities. In end, we conclude the challenges existed in this field and propose the potential opportunities in aspects from the discovery of novel *p*-type 2D semiconductors, their controlled mass preparation, compatible engineering with silicon production line, high-*κ* dielectric materials, to integration and applications of *p*-type 2D semiconductors and their heterostructures in electronic and optoelectronic devices. Overall, we believe that this review will guide the design of preparation systems to fulfill the controllable growth of *p*-type 2D semiconductors with high quality and thus lay the foundations for their potential application in electronics and optoelectronics.

## Highlights

- Compared to the *n*-type two-dimensional (2D) semiconductors, the family of *p*-type 2D semiconductors is relatively small, which limits the broad integration of 2D semiconductors in potential applications. Here, the discovery and preparation of *p*-type 2D semiconductors are very important and meaningful.
- This review presents a timely and in-depth overview on the preparation and applications of *p*-type 2D semiconductors, which would help the related researchers to grasp the dynamics of this field and thus lay the foundations for their potential application in electronics and optoelectronics.

## Graphical abstract

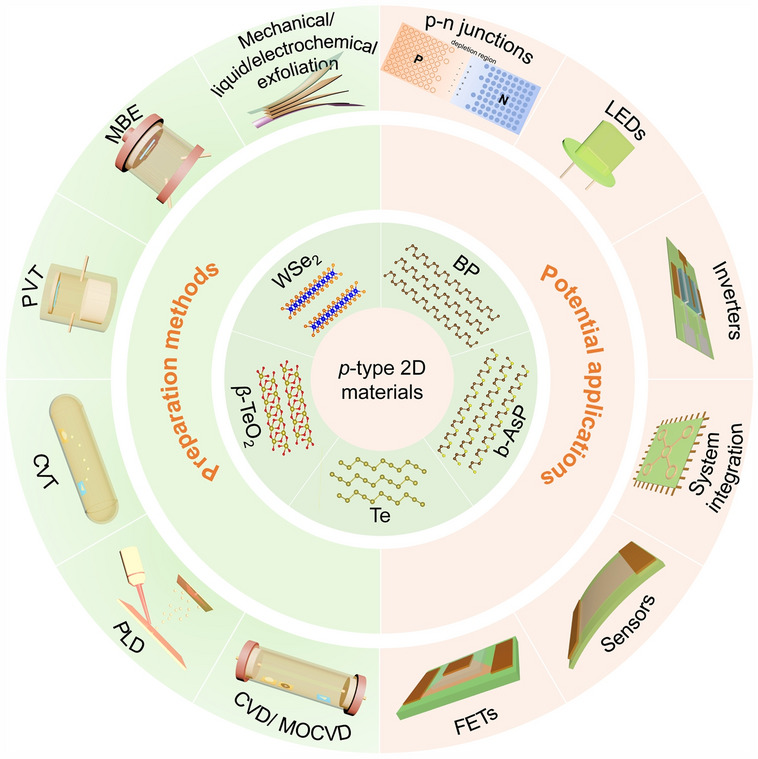

## Introduction

Materials are the basis for the development of science and technology, driving the social progress and civilization of human beings. In the face of the ever-increasing demand for miniaturization of devices, silicon-based integrated circuits are approaching their limitation due to the short-channel effect, thermal effect, and manufacturing costs [1]. The exploration of new channel materials that are compatible with silicon-based technology has become one of the most popular research fields, and the low-dimensional material is perceived as the key solution to continue *Moore*’s law [2]. Since the first report of monolayer graphene exfoliated by *Scotch* tape in 2004 [3], two-dimensional (2D) materials have gained widespread attention, among which 2D semiconductors exhibit important advantages over the traditional silicon and III–V semiconductors (*e.g*., GaAs and GaN). For example, the atomic-level thickness, tunable bandgaps, dangling bond-free surfaces, high carrier mobility, and many other superior properties [4], all make 2D semiconductors promising candidates in the electronics [5–10] and optoelectronics [11–19]. Their preparation and application in large-scale 2D integrated electronics are proceeding continuously.

Both the *n*-type and *p*-type materials are the basic building blocks of electronic and optoelectronic devices. However, due to the strong electron doping impurities and inherent structural defects of interface charges [20], most 2D semiconductors are *n*-type. In addition, the Fermi level pinning at the metal/2D semiconductor interface leads to a higher Schottky barrier for hole injection [21], which greatly hinders the *p*-type conduction in 2D semiconductors and their practical applications such as complementary logic circuits [1], inverters [22], field-effect transistors (FETs) [23], and light-emitting diodes (LEDs) [24]. Moreover, *p*-type 2D semiconductors are the essential components in functional van der Waals (vdW) heterostructures, which show exotic properties and promising device performance beyond of the capabilities of existing materials [25, 26]. Therefore, the discovery of *p*-type 2D semiconductors and realization of their controllable preparation with large size and high quality are critical for the continuing development of this field.

**Fig. 1:**
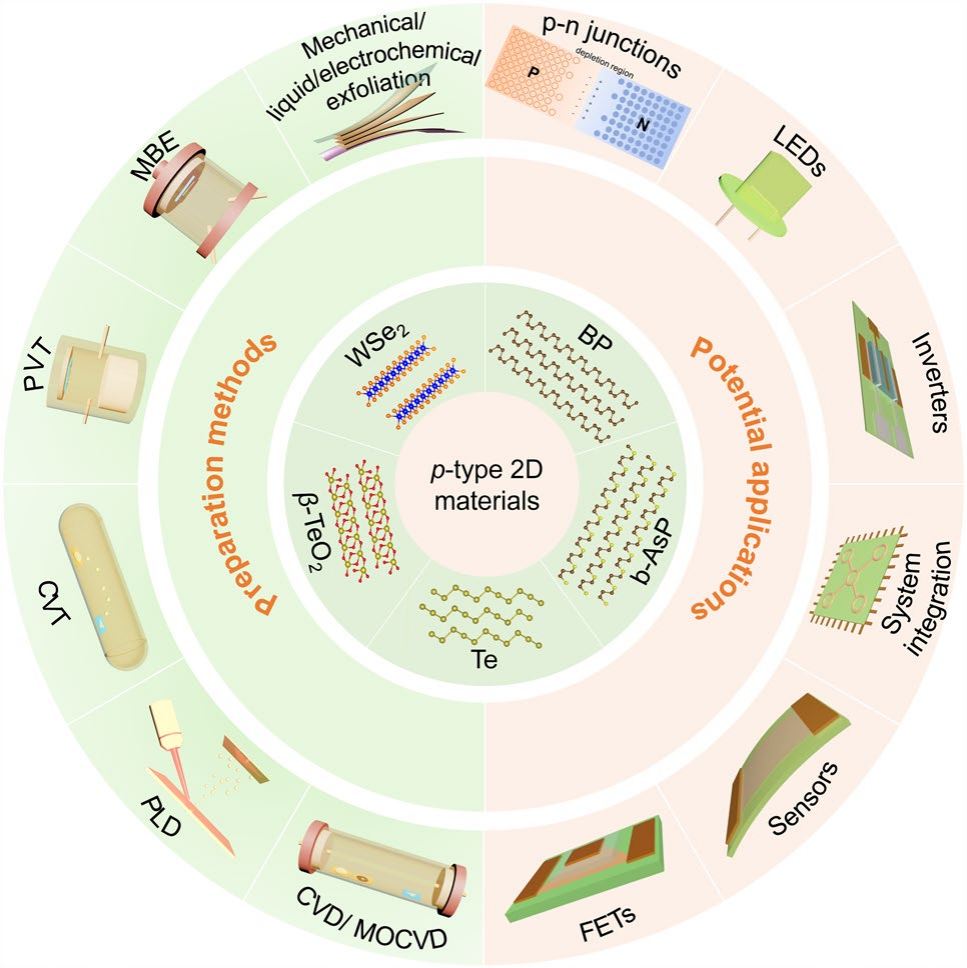
A summary of *p*-type 2D semiconductors, their preparation methods, and potential applications

In this review, we summarize the candidates of *p*-type 2D semiconductors. Then, we highlight the main strategies for the controlled preparation of *p*-type 2D semiconductors and recent progresses achieved in this field. We also introduce some applications of *p*-type 2D semiconductors to show their future prospects. Finally, we put forward the main challenges existed in this field and point out the potential research directions of *p*-type 2D semiconductors (Fig. 1).

## Preparation of *p*-Type 2D Semiconductors

In the past few years, the search for *p*-type 2D semiconductors has succeeded in identifying only a few candidates such as WSe_2_, BP, black arsenic phosphorus (b-AsP), *α*-MnS, Te, *β*-TeO_2_, *h*-TiO_2_, SnO, Ni_x_O, Cu_x_O, MoSi_2_N_4_, and pentacene, as summarized in Table 1. In this part, we will focus on recent progress achieved in the preparation of these *p*-type 2D materials and briefly introduce the drawbacks of these methods.

**Table 1:** A Summary of the Discovered *p*-Type 2D Semiconductors

Material	Bandgap (eV)	Method	Application	Carrier mobility (cm^2^ V^−1^ s^−1^)	*I*_on_/*I*_off_	Refs.
WSe_2_	1.64	Mechanical exfoliation	FET	250	> 10^6^	[27]
	1.5	Liquid exfoliation	FET	NA	10^2^	[28]
	1.64	PVT	FET	90	10^6^	[29]
	1.64	CVD	FET	143	9 × 10^6^	[30]
BP	0.3	PLD	FET	213	10^3^	[31]
b-AsP	0.15–0.3	CVT	FET	110	1.9 × 10^3^	[32]
*α*-MnS	2.7	CVD	FET	0.1	> 10^6^	[33]
Te	0.5	Thermal evaporation	TFT	35	10^4^	[34]
*β*-TeO_2_	3.7	Oxidation	FET	232	10^6^	[35]
*h*-TiO_2_	2.35	Oxidation and mechanical exfoliation	FET	950	10^6^	[36]
SnO	2.7	Magnetron sputtering	TFT	1.4	3.10 × 10^2^	[37]
Ni_x_O	NA	Solution	TFT	25	10^5^–10^6^	[38]
Cu_x_O	NA	Solution	TFT	0.07–0.28	10^3^–10^6^	[38]
MoSi_2_N_4_	1.94	CVD	FET	NA	4 × 10^3^ at 77 K	[39]
Pentacene	NA	PVT	FET	5.6	10^5^	[40]

### Top–Down Methods

Controllable preparation of materials is the first prerequisite to meet the increasing requirements of many potential high-performance devices. Up to now, *p*-type 2D semiconductors have been prepared by top–down and bottom–up methods. For top–down methods, *p*-type 2D semiconductors are prepared from their bulk counterparts by disassembling the vdW layers through micromechanical, liquid-phase exfoliation, or electrochemical exfoliation routes. Actually, the top–down method becomes a universal methodology for the preparation of a series of *p*-type 2D semiconductors.

#### Micromechanical Exfoliation

**Fig. 2:**
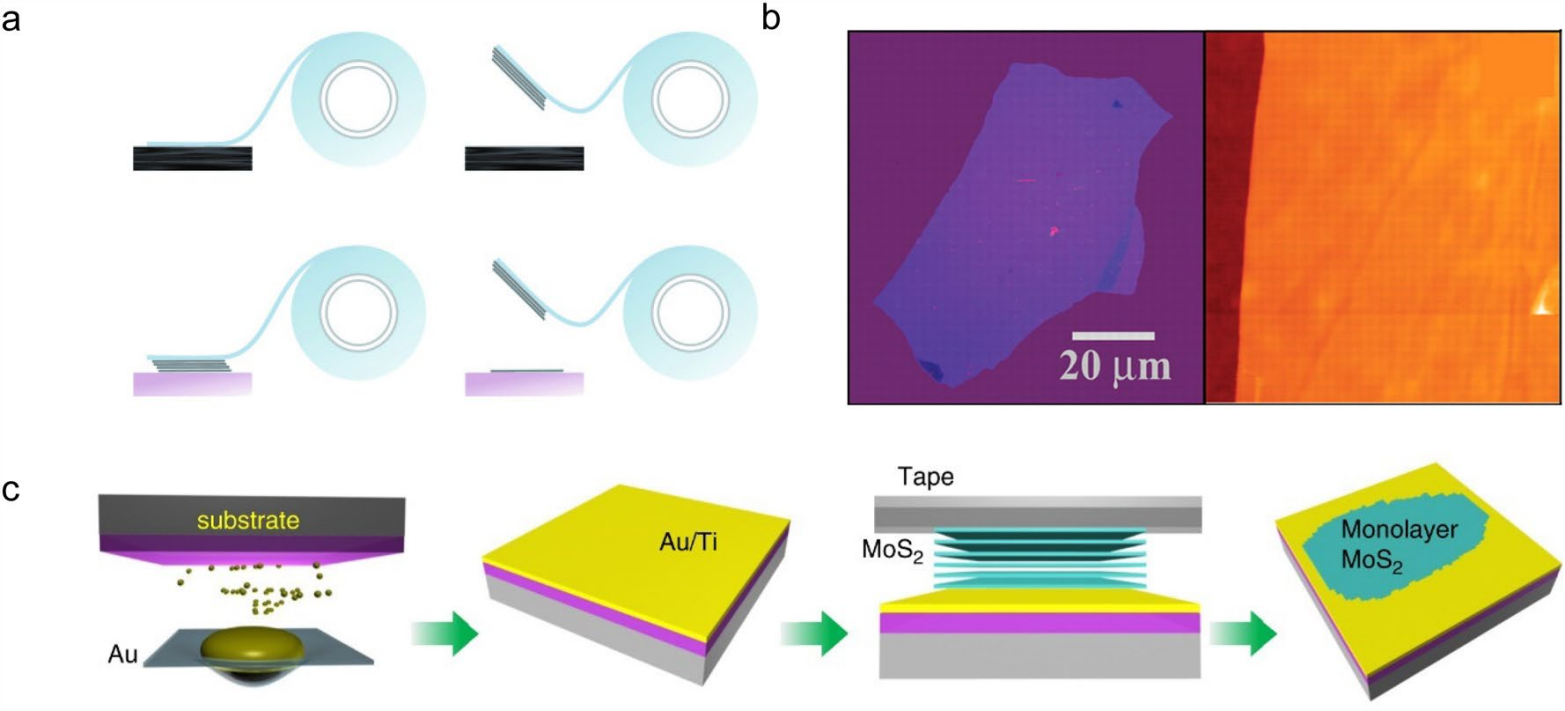
Micromechanical exfoliation method for the preparation of 2D materials. **a** Micromechanical exfoliation method for the preparation of graphene [41]. **b** Photograph of a relatively large multilayer graphene flake with a thickness of 3 nm on the SiO_2_/Si substrate [3]. **c** Micromechanical exfoliation method with the assistance of thin Au film [42]

The micromechanical exfoliation of monolayer graphene using the *Scotch* tape opens an avenue for the study of 2D materials (Fig. 2a and b) [3, 41]. It is accessible to exfoliate graphene and other 2D materials from their bulk counterparts by hand-tearing the adhesive tape to get high-quality 2D materials with thickness of monolayer. In a recent study, Huang et al*.* developed a contamination-free, one-step, and universal exfoliation method with the assistance of Au thin film [42]. The thin Au film can form quasi-covalent bonds with two-dimensional materials, and the interaction force is larger than the interlayer vdW force in the material. So, with the help of Au as the medium layer, large monolayer samples were efficiently cleaved without affecting their intrinsic physical properties (Fig. 2c). They obtained more than 40 types of single-crystalline monolayer 2D materials with millimeter size, such as elementary 2D crystals, metal dichalcogenides, transition metal dichalcogenides (TMDCs), magnets, and superconductors. The micromechanical exfoliation method can also cooperate with the transfer and stacking technique to fabricate 2D heterostructures and twisted structures [43–45]. However, it is still difficult to control the number of layers and yield via micromechanical exfoliation, and it is highly dependent on the experimenters’ skill, which makes it not suitable for the large-scale preparation of 2D materials. In addition, the parent bulk crystal is the essential raw materials for the micromechanical exfoliation method. It is not feasible for the exploration of novel *p*-type materials without known bulk counterparts, such as MoSi_2_N_4_ [39].

#### Liquid-Phase Exfoliation

**Fig. 3:**
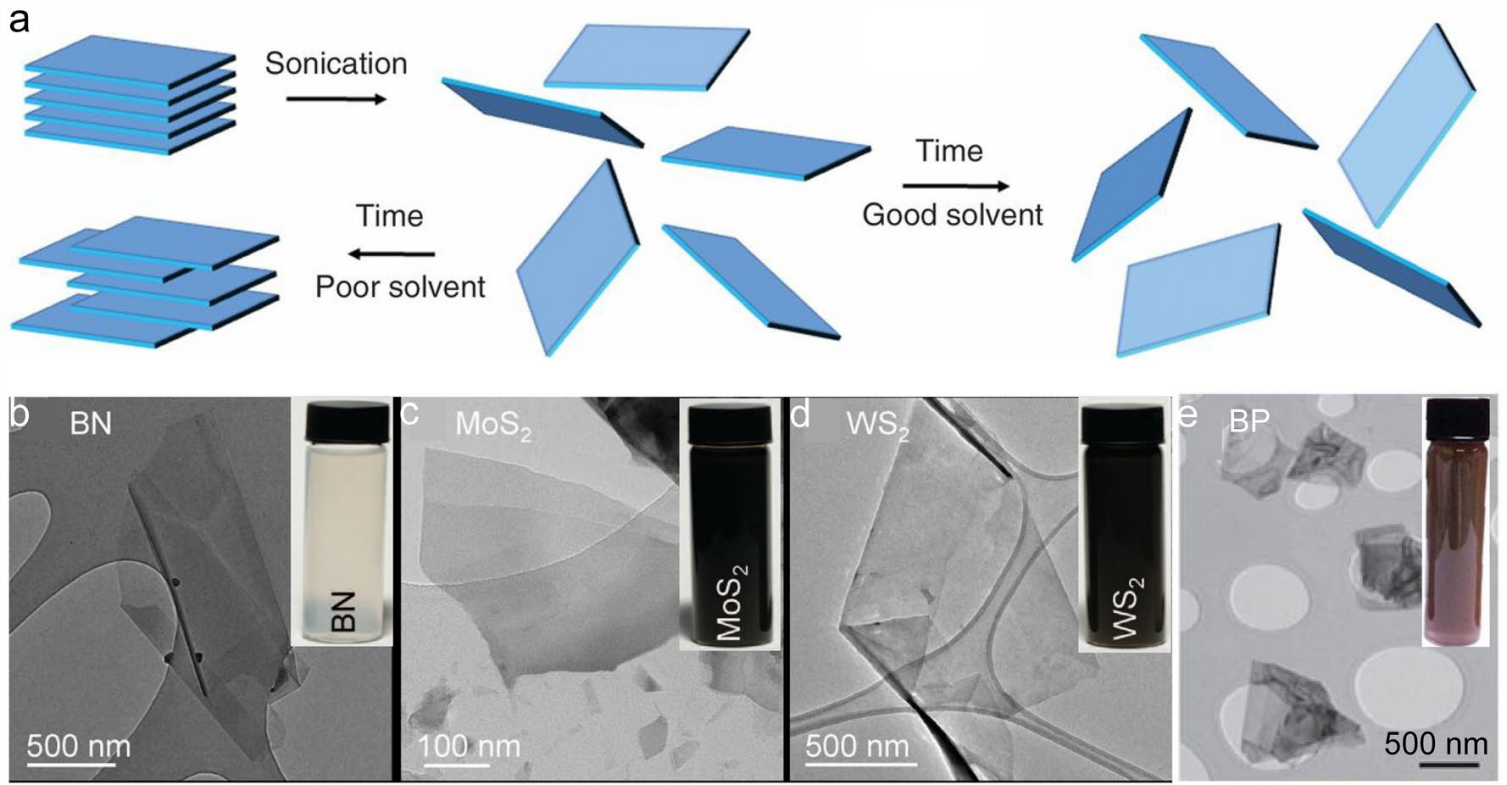
Liquid-phase exfoliation method for the preparation of 2D materials. **a** Schematic of the liquid-phase exfoliation method [46]. **b** Universality for the preparation of various 2D nanosheet inks by ultrasonic dispersion [47, 48]

Liquid-phase exfoliation is another top–down method to prepare 2D materials, and it can be divided into metal-ion intercalation, oxidation exfoliation, and ultrasonic dispersion exfoliation according to the driving force*.* For example, Coleman et al*.* ultrasonically treated the bulk materials for a long time in organic solvents such as isopropanol (IPA) and N-methyl 2-pyrrolidone (NMP) (Fig. 3a) [46] and obtained a series of 2D nanosheet inks, including insulating h-BN, *n*-type MoS_2_, WS_2_, and *p*-type BP (Fig. 3b–e) [47, 48]. These as-prepared inks could be easily printed to form 2D thin films and thus to fabricate thin-film transistors (TFTs). For example, Kelly et al*.* fabricated the network transistors by using the suspensions of MoS_2_, MoSe_2_, WS_2_, and WSe_2_ nanosheets with the mean lateral size and thicknesses in the proper ranges, *i.e.*, 330–380 nm and 13–17 layers, respectively. The all-printed and vertically stacked transistors contained the graphene source, drain, as well as gate electrodes, the TMDC (*e.g.*, MoS_2_ and WSe_2_) channels, and the BN separator under the electrolytic gating. These devices showed the on/off current ratio of 600, transconductance of > 5 millisiemens, and mobility of > 0.1 cm^2^ V^−1^ s^−1^ [49]. This scalable printing method for the fabrication of devices provides one promising method for next-generation large-scale electronic applications. Overall, the biggest advantage of liquid-phase exfoliation is that it can realize the scalable mass production of 2D materials and their composites, accelerating their applications in real life. However, this method consumes lots of solvents, and the sample surfaces are easy to be contaminated. Besides, the sample size is still small, and the number of layer is difficult to control via liquid-phase exfoliation. So, further treatment, *e.g*., density gradient centrifugation (DGU), is needed to improve the uniformity of the domain size to meet the application requirements of electronic and optoelectronic devices [50, 51].

#### Electrochemical Exfoliation

**Fig. 4:**
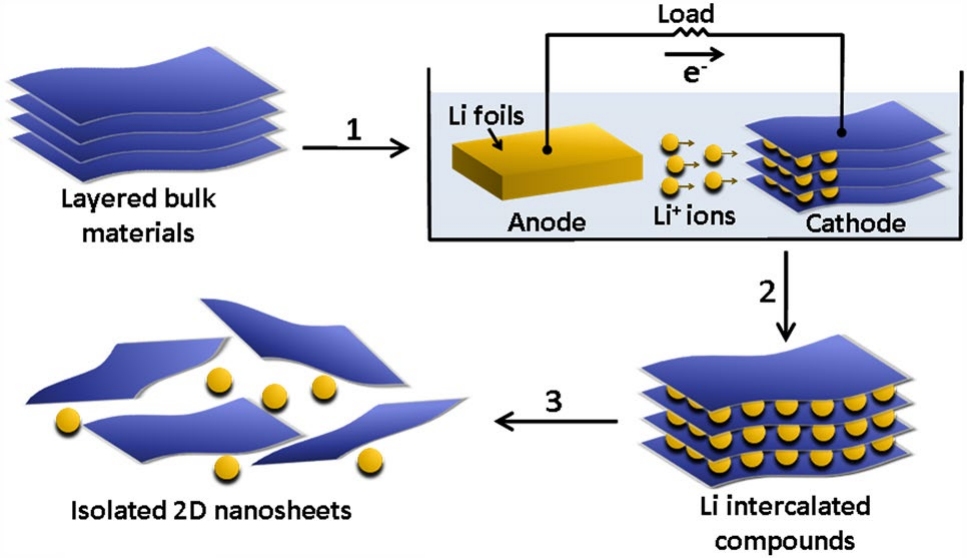
Schematic of the electrochemical exfoliation method to prepare 2D materials [52]

The above-mentioned liquid-phase exfoliation method relies on the solvent, *e.g.,* IPA and NMP, to minimize the energy of exfoliation. It caused many difficulties to realize the mass production of single-layer 2D materials with high yields. In order to overcome this problem, Zeng et al. developed an effective method to prepare single-layer 2D materials with high yields by using a controllable lithiation process [52]. They used the layered bulk materials as the cathode in an electrochemical set-up (Fig. 4), and then, lithium ions were intercalated in these bulk materials in a well-controlled manner during the discharge process to get single-layer 2D materials. Here, the bulk layered materials were incorporated in a test cell as the cathode (Step 1). The lithium foil was used as the anode to provide lithium ions to produce the intercalated compounds (Step 2). With the subsequent ultrasonication of these intercalated compounds in water or ethanol solution (Step 3), single-layer 2D nanosheets were obtained with high yields. The reasonable mechanism is that: The Li^+^ ions expanded the interlayer spaces of the vdW materials, and then, the metallic Li (after insertion Li^+^ ions were reduced by electrons during the discharge process) reacted with water to produce lots of H_2_ bubbles, which further expanded the interlayer spaces of the vdW materials. Eventually, with the sufficient agitation provided by the following ultrasonication process, isolated 2D nanosheets with single layer were obtained. Overall, this method provides a new sight to exfoliate 2D materials and has been widely used to prepare ultrathin WSe_2_ and BP nanosheets [53, 54]. In the next stage, we need to improve the efficiency of the electrochemical exfoliation process and make it more environmentally friendly.

### Bottom–Up Method

Bottom–up method, including molecular beam epitaxy (MBE), physical vapor transport (PVT), pulsed laser deposition (PLD), chemical vapor transport (CVT), chemical vapor deposition (CVD), and metal–organic CVD (MOCVD), is another universal approach to prepare *p*-type 2D semiconductors. Compared with the top–down method mentioned above, these bottom–up method shows the great potential to prepare large-area 2D materials with electronic-grade quality and reasonable cost. In this part, we will review current progresses achieved in the growth of *p*-type 2D semiconductors via these bottom–up method as well as their shortcomings.

#### MBE

MBE involves a vapor deposition process in an ultra-high vacuum (around 10^–6^ mbar) chamber, in which the molecular beam of precursors is sprayed onto the substrate surface to grow thin films. The films are epitaxial and layer-by-layer grown along the crystal axis direction of the substrate to achieve high quality. The advantages of this method include: (i) highly clean surface and controllable thickness (atomic level) and (ii) precisely controlled composition and doping concentration owing to the tunable dosage ratio of reactants.

**Fig. 5:**
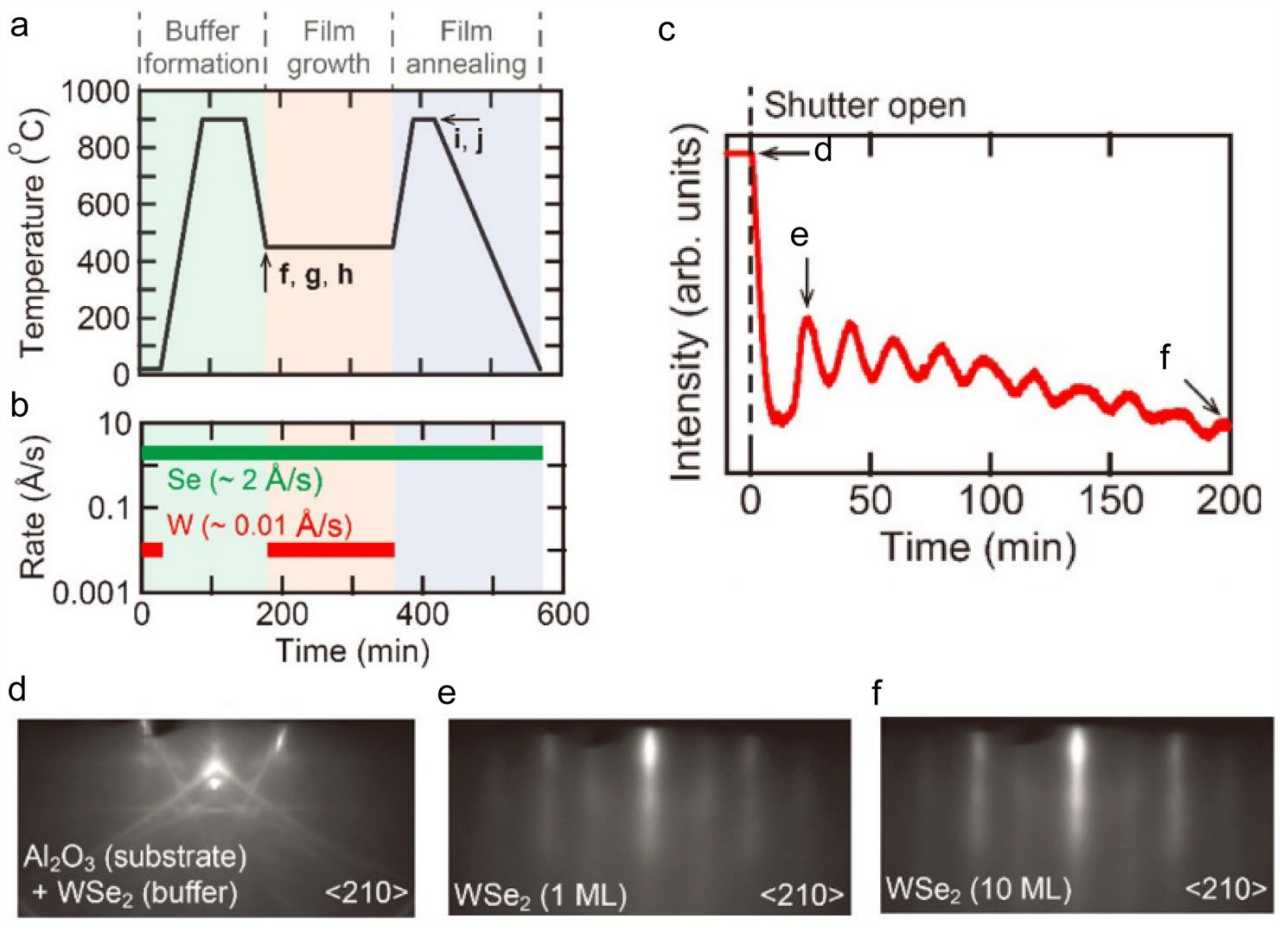
MBE growth of *p*-type 2D semiconductors. **a** Schematic diagram of the growth process. **b** Typical evaporation rates of W and Se at each stage of the film growth. **c** Time evolution of the RHEED intensity recorded during growth of WSe_2_ film. **d-f** RHEED patterns along ⟨210⟩ azimuth of the substrate taken at each position of the as-grown WSe_2_ film [55]

Nakano et al*.* reported the layer-by-layer epitaxial growth of WSe_2_ thin films on insulating Al_2_O_3_ (001) substrates by MBE and demonstrated the ambipolar transistor operation realized by electrolyte gating. They chose Al_2_O_3_ (001) single crystal as the growth substrate due to its hexagonal lattice facilitated the c-axis orientated growth of WSe_2_. Besides, well-defined regular steps and terraces were formed on the atomically flat surface of Al_2_O_3_ after being annealed in air and thus facilitated the growth of high-quality WSe_2_ thin films [55]. Before the film deposition, a buffer layer consisting of W and Se with thickness less than a monolayer was formed at room temperature, followed by the annealing at 900 °C for 1 h. Then, the substrate was cooled down to 450 °C, and the main growth process was conducted. When the growth process finished, the sample was annealed again at 900 °C for half an hour to improve the crystallinity of as-grown thin film and then cooled down to room temperature (Fig. 5a and b). In addition, using the real-time reflection high-energy electron diffraction (RHEED) (Fig. 5c–f), some useful information was acquired, such as the growth rate, time evolution of crystallinity, surface roughness of as-grown thin films, in-plane crystallographic orientation, and its relationship to the sapphire substrate during the MBE process, from which the authors confirmed that the WSe_2_ thin film was grown on Al_2_O_3_(001) substrate following a layer-by-layer mode and consistent with the previous work [56]. However, there are some problems with MBE to grow 2D TMDCs, including the complicated deposition process, expensive facility cost, strict substrate matching symmetry, and low growth rate. We need to find ways to address these issues to make MBE more useful and universal in the preparation of *p*-type 2D semiconductors.

#### PVT

**Fig. 6:**
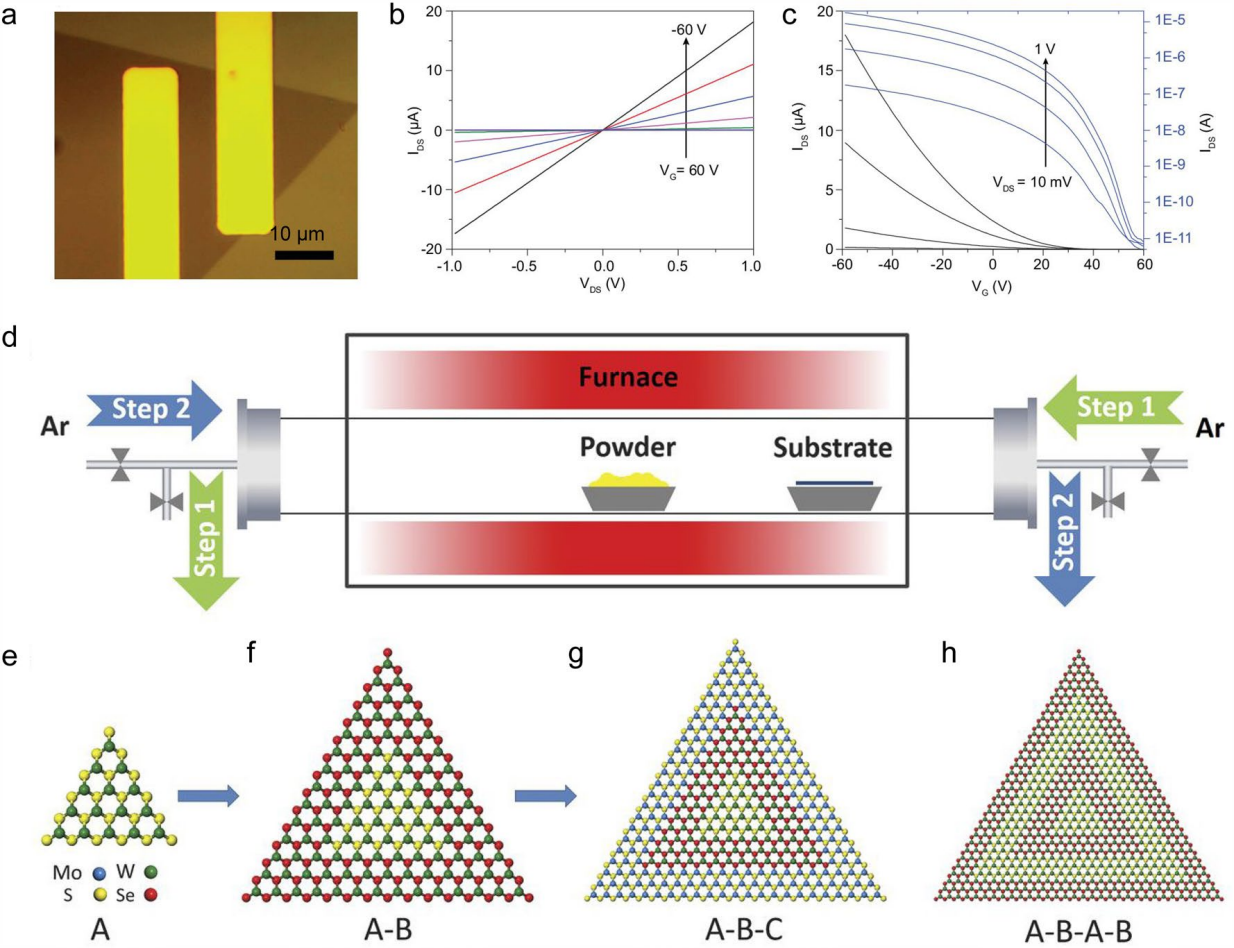
PVT growth of *p*-type 2D semiconductors. **a** OM image of a monolayer WSe_2_ transistor with two transferred Pt electrodes supported on the Si/SiO_2_ substrate. **b** *I*_d_–*V*_d_ output characteristics of a typical WSe_2_ transistor. **c** *I*_d_–*V*_g_ transfer characteristics at *V*_d_ = 10 mV, 100 mV, 500 mV, and 1 V [29]. **d** Schematic of a modified PVT system using reversed gas flow for the epitaxial growth of various 2D heterostructures. Evolution of the growth product from **e** a monolayer seed A, **f** A-B heterostructures, **g** A-B-C multiheterostructures, and **h** finally to A-B-A-B superlattices [59]

PVT synthesis of 2D materials involves the sublimation of target materials at high temperature, the transport of the precursor vapors to the condensate areas to form the saturated steam, and finally the nucleation and growth of single crystals [57]. It is quite feasible to prepare thin inorganic and organic films on substrate. For example, Wu et al*.* first reported the PVT growth of monolayer MoS_2_ single crystals with 25 μm in domain size on various substrates (*e.g.*, SiO_2_, sapphire, and mica). This method is simple and reliable, and the optical quality of the as-grown crystals is extremely high. The valley polarization approaches 35% even at room temperature, suggesting a virtual absence of defects [58]. Zhang et al*.* reported a modified PVT method with the controllable reverse flow and realized the rapid growth of monolayer WSe_2_ single crystals with large domain size. The size of the as-grown monolayer WSe_2_ reached 450 μm within 10 s, and the highest lateral growth rate reached 45 μm s^−1^. The FET based on the as-grown WSe_2_ also exhibited excellent electronic performance with a carrier mobility of up to 90 cm^2^ V^−1^ s^−1^ (Fig. 6a-c) [29]. Furthermore, they demonstrated that this modified PVT method was universal to grow diverse 2D lateral heterostructures (*e.g.*, WS_2_-WSe_2_ and WS_2_-MoSe_2_), multiheterostructures (*e.g.*, WS_2_-WSe_2_-MoS_2_ and WS_2_-MoSe_2_-WSe_2_), and superlattices (*e.g.*, WS_2_-WSe_2_-WS_2_-WSe_2_-WS_2_) (Fig. 6d–h). Transmission electron microscope studies clearly showed the atomically sharp compositional transition at their interfaces. And electrical transport studies of the WSe_2_-WS_2_ lateral junctions showed well-defined diode characteristics with a rectification ratio of 10^5^ [59]. These studies provide an innovative pathway to grow high-quality TMDC single crystals, heterostructures, and superlattices via PVT.

Besides inorganic *p*-type 2D semiconductors, PVT can also fulfill the controllable synthesis of organic *p*-type 2D semiconductors. For example, Arabi et al*.* reported the controlled growth of ultrathin 2D pentacene crystal via a nano-seed-assisted PVT method. The size, thickness, and density of pentacene crystals were systematically optimized the growth parameters, they obtained large and ultrathin 2D pentacene crystals. The pentacene-based FET showed a clear *p*-type transfer behavior and a high hole mobility of 5.6 cm^2^ V^−1^ s^−1^ [40]. It is one of the most useful and *p*-type organic molecular crystals for the construction of organic electronics.

#### CVT

CVT is usually used to grow bulk single crystals. In detail, the raw material A and transport agent B (*e.g.*, I_2_) are mixed in the quartz container and then react to produce the volatile product C at a high temperature and high pressure (HTHP) following the chemical equilibrium:

$$ A_{(s)} + \, B_{(g)} \leftarrow \to \, C_{(g)} $$

The equilibrium constants of the reaction vary with the temperature. Once the generated gaseous substance C is transported from the initial side to the other side of the container, the equilibrium will move to the opposite direction, *i.e*., *C*_(*g*)_ → *A*_(*s*)_ + *B*_(g)_. During this process, the raw material A will be purified and deposited to form highly crystalline single crystals. So far, many commercial companies such as *2D Semiconductors* and *HQ Graphene* have obtained various bulk materials (*e.g.*, BN, MoS_2_, WSe_2_, BP, and Bi_2_O_2_Se) via CVT.

**Fig. 7:**
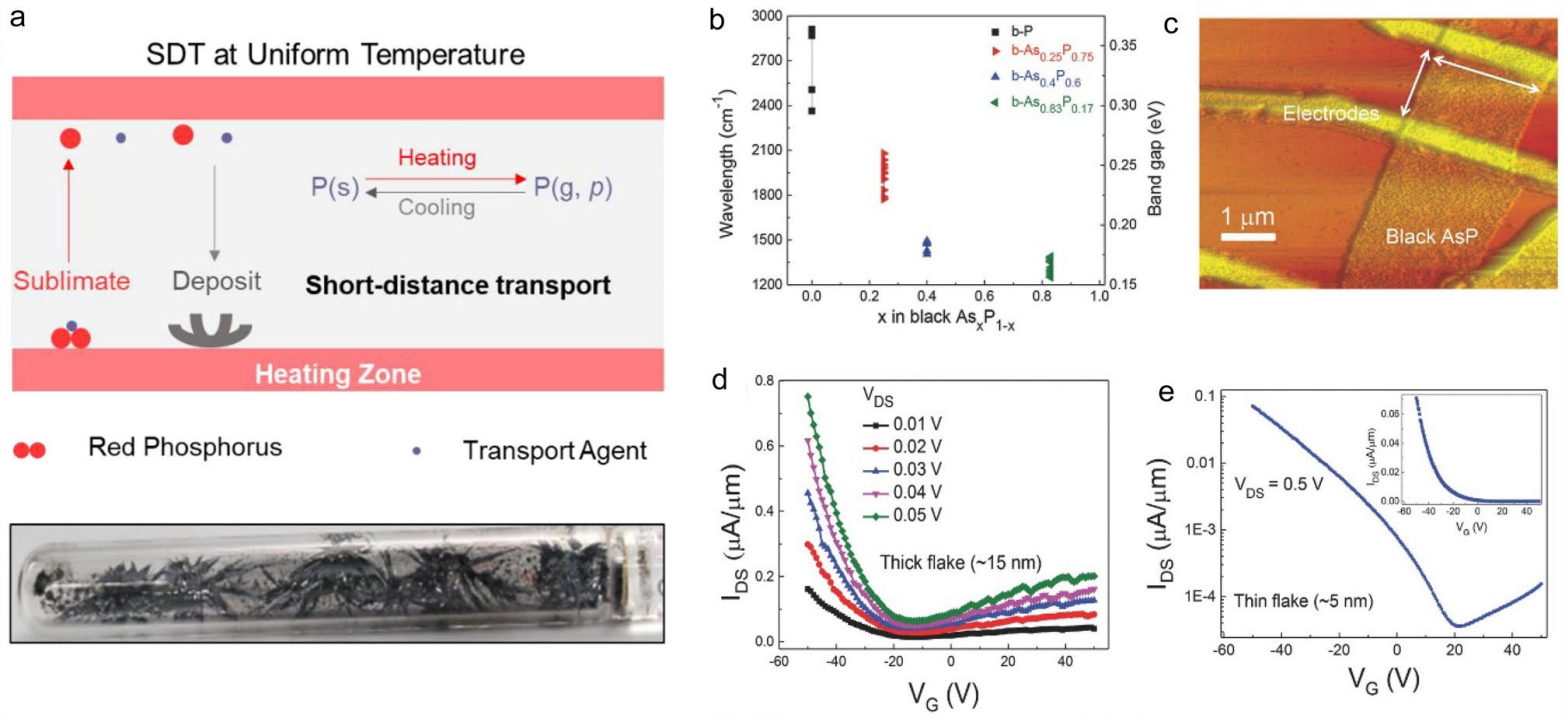
CVT growth of *p*-type 2D semiconductors. **a** Scheme of the SDT method to grow BP and the photograph of the as-grown sample [60]. **b** Summary of the x-dependent bandgaps of b-As_*x*_P_1-*x*_ (thickness > 30 nm). **c** Typical atomic force microscopy (AFM) image of the b-As_*x*_P_1-*x*_-based FET. **d** Transfer curves of a thick b-As_0.83_P_0.17_ flake with 15 nm in thickness. **e** Transfer curve of a thin b-As_0.83_P_0.17_ flake with 5 nm in thickness in logarithmic scale and linear scale (inset) [32]

Recently, Liu et al*.* reported an efficient short-distance transport (SDT) growth approach which solved the issue of low yield in the traditional CVT growth of BP, achieving the highest yield of 98% as well as the high quality (Fig. 7a). Besides, various heteroatoms such as As, Sb, Bi, Se, and Te were successfully doped into the BP lattice to modify its electronic structures including bandgap, work function, and energy band position [60]. Using the mineralizer-assisted short-way CVT, Liu et al. also realized the controllable growth of black arsenic–phosphorus with different compositions (b-As_x_P_1-x_, with x in the range of 0–0.83) by adjusting the adding amount of the As dopant (Fig. 7b). Owing to the widely tunable chemical compositions, the as-grown layered b-As_x_P_1-*x*_ showed widely tunable bandgaps and optical properties. b-As_*x*_P_1-*x*_ covered long wavelengths down to around 8.27 µm (0.15 eV), which was located in the long-wavelength infrared regime. The FET based on the 15-nm thick b-As_0.83_P_0.17_ flake showed a typical *p*-type transfer behavior with a hole mobility of 110 cm^2^ V^−1^ s^−1^ (Fig. 7c and d). And another device based on a thin (5 nm) b-As_0.83_P_0.17_ showed an on/off current ratio of 1.9 × 10^3^ (Fig. 7e) [32].

However, the feasibility of CVT in the preparation of monolayer 2D materials should be extended. Now, there are only several materials including MoS_2_ and WS_2_ can be grown in their few- or monolayer forms via CVT. The main challenges that hinder the development of CVT include precursor concentration, complicated growth process, long growth time, low efficiency, and high cost. More efforts are needed to overcome these drawbacks to make CVT more efficient in the synthesis of various *p*-type 2D semiconductors and other 2D materials with monolayer.

#### PLD

**Fig. 8:**
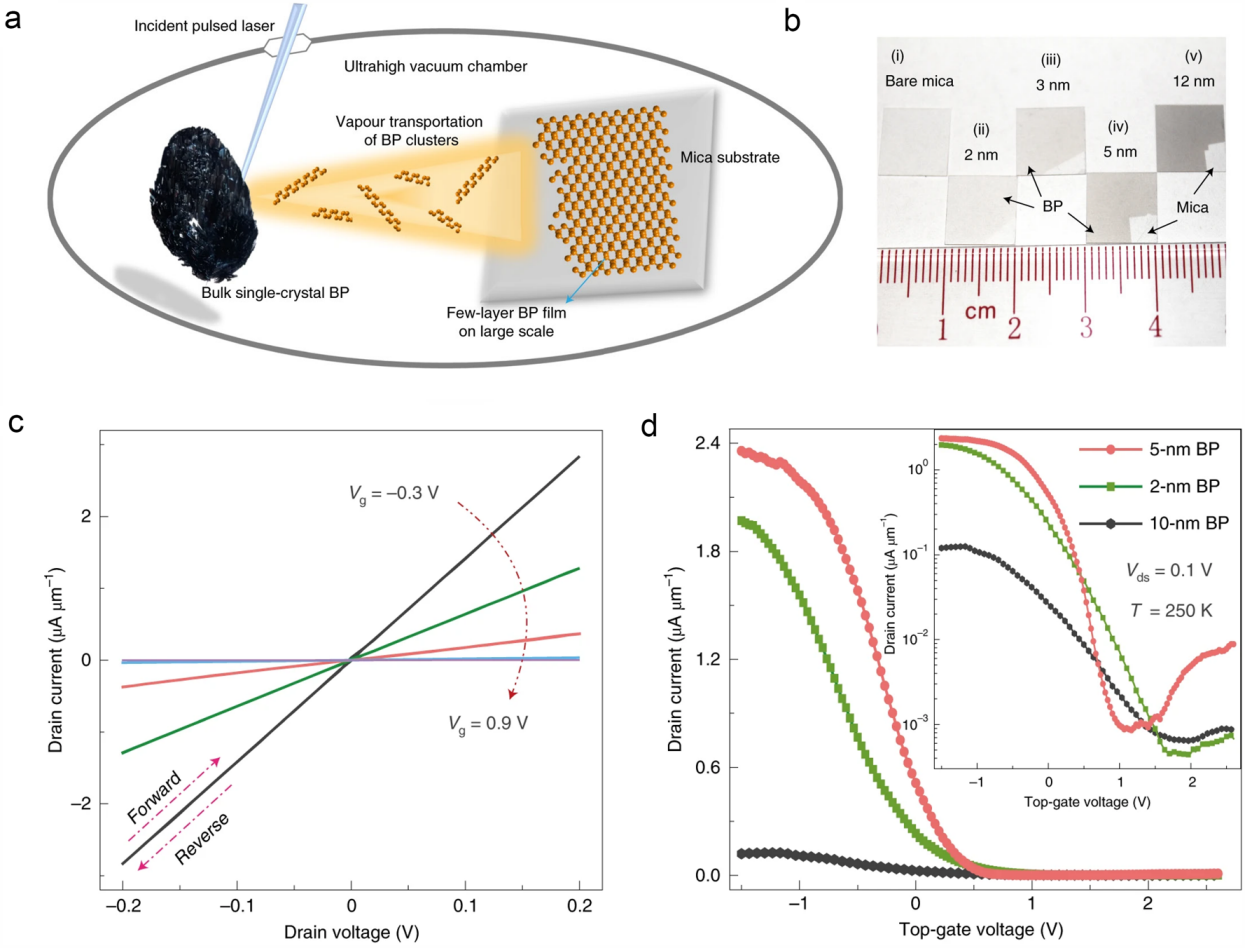
PLD growth of *p*-type 2D BP thin film. **a** Schematic of the PLD process used for the growth of few-layer BP films, **b** photographs of bare mica (i) and as-grown centimeter-scale BP films with different thicknesses (ii-v). **c** *I*_d_–*V*_d_ curves of a FET based on a 5-nm thick BP film under different gate voltages at 250 K. **d** Transfer characteristics of FETs based on BP films with thicknesses of 2, 5, and 10 nm in linear scale at 250 K, the inset shows the same results in logarithmic scale [31]

PLD uses high-power pulsed laser to bombard the target material to facilitate the deposition of the final product with desired stoichiometry, phase, film thickness, morphology, and composition by varying the deposition parameters in the vacuum chamber [61]. Recently, Wu et al*.* reported a pioneering work which realized the synthesis of high-quality few-layer BP film on the centimeter scale via PLD in a controlled manner (Fig. 8a and b). According to the molecular dynamic simulation, the authors found that the high-power pulsed laser facilitated the formation of large BP clusters within the transported physical vapor and thus led to the reduction of the formation energy of BP to enable the growth of large-scale few-layer BP film. The centimeter-scale FET arrays based on the as-grown BP film (5 nm in thickness) showed high carrier mobilities of 213 and 617 cm^2^ V^−1^ s^−1^ at 295 and 250 K, respectively (Fig. 8c and d) [31]. This work provides a promising approach to achieve high uniformity throughout the whole large-scale film, laying the foundation for the further development of BP-based devices. Nevertheless, PLD has its own drawbacks. The product cannot strictly follow the stoichiometric ratio, and some clusters may appear on the surface of the film. All these issues will significantly degrade the performance of the as-grown films.

#### CVD

CVD has been used to grow lots of 2D materials. However, due to the use of multiple precursors and the complicated vapor-phase growth process, there are still many problems remained in the CVD growth of high-quality 2D materials, such as the non-uniform distribution of as-grown domains, high-concentration defects and vacancies, and the complicated growth mechanism. All these problems should be carefully dealt with to achieve the precise control of the growth process of 2D materials [4, 62, 63].

**Fig. 9:**
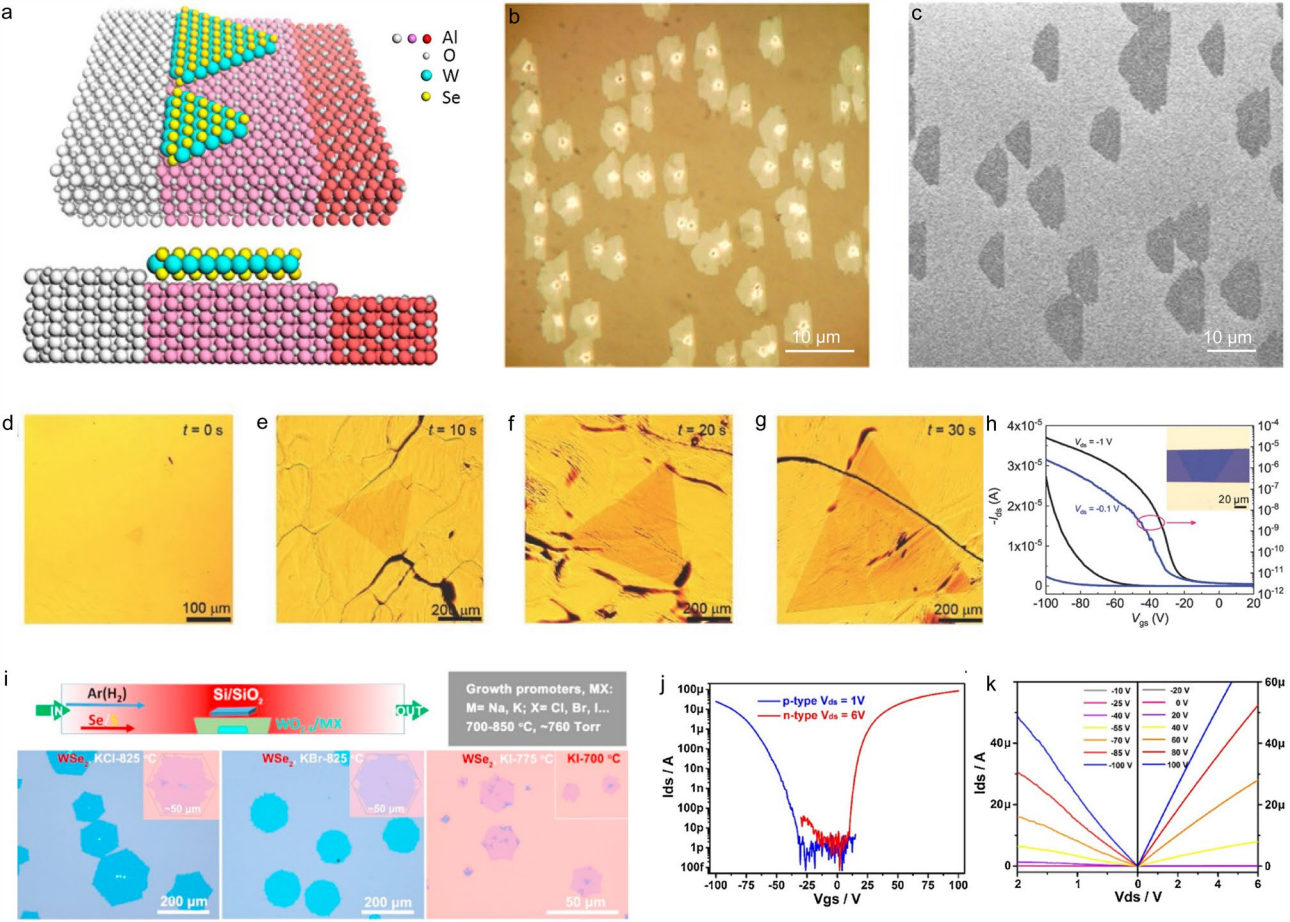
CVD growth of *p*-type 2D semiconductors. **a** Schematic of the step-edge-guided nucleation and growth of aligned WSe_2_ on a C-plane sapphire substrate and **b, c** its growth results [65]. **d–g** Typical optical images of single-crystal monolayer WSe_2_ domains grown on an Au foil at time *t* = 0, 10, 20, and 30 s. **h** Transfer characteristics of a monolayer WSe_2_ FET on the SiO_2_/Si substrate [30]. **i** Schematic illustration of the CVD-grown TMDCs with the addition of alkali metal halides. **j** Transfer characteristics of *p*-type (blue) and *n*-type (red) WSe_2_ FETs, *p*-type and *n*-type devices were prepared using Pd/Au and Ag/Al/Au as contacts, respectively. **k** Corresponding output curves of the WSe_2_ devices: *p*-type (left panel) and *n*-type (right panel) [71]

Pre-treatment of the substrates, *e.g.*, annealing of the substrate, is one of the most useful methods to promote the controllability of CVD process. After the annealing treatment of the substrate, specific dominant crystal plane is exposed to facilitate the epitaxial growth of 2D materials, and this will make the as-grown 2D domains orient in the same direction, which is very critical for the synthesis of wafer-scale single-crystal film. Without this treatment, the as-grown single domains will be randomly oriented. When they merge together, a polycrystalline film forms with lots of grain boundaries embedded in the film between adjacent domains, which greatly degrade the mechanical, electrical, and thermal properties of the as-grown 2D polycrystalline film [64]. In contrast, the well-aligned single-crystal 2D domains can be seamless stitched together and thus form wafer-scale monocrystalline films, which will boost the performance of the electronic or optoelectronic devices based on the monocrystalline film. As a proof-of-concept, several attempts have been made to realize of the seamless growth 2D materials. For example, Liu et al*.* proposed a step-edge-guided nucleation mechanism and achieved the aligned growth of WSe_2_ on C-plane (0001) sapphire substrate (Fig. 9a). After annealing at a high temperature of > 950 °C for several hours, atomic steps formed on the sapphire surface and served as active nucleation sites to guide the formation of well-aligned WSe_2_ nucleus. With the increasing growth time, WSe_2_ tended to follow the layer-by-layer growth mode, and finally, well-aligned few-layer domains formed as revealed by the optical microscopy (OM) and scanning electron microscopy (SEM) images shown in Fig. 9b and c. This work provides an efficient method to fulfill the oriented growth of 2D WSe_2_ and adds fresh knowledge on the growth mechanism of WSe_2_. This method now has been used to grow other 2D TMDCs in wafer scale [65–68].

Except for the annealing of the substrates, using the catalytically active substrates to improve the growth controllability of 2D materials is another choice. Generally, the decrease in the active surface energy of the substrate will benefit the nucleation and growth of 2D materials during the CVD process. For example, Gao et al*.* reported the catalytic growth of monolayer WSe_2_ on Au foil with a high growth rate of 26 μm s^−1^, which is 2–3 orders of magnitude higher than those reported in other works [69, 70]. Millimeter-scale monolayer single-crystalline WSe_2_ domains were achieved within 30 s while large continuous films were obtained within 60 s (Fig. 9d-g) [30]. The FET based on this monolayer WSe_2_ showed the hole mobility of 143 cm^2^ V^−1^ s^−1^ and on/off current ratio of 9 × 10^6^ at room temperature, which is comparable to that of the mechanically exfoliated samples (Fig. 9h). Density functional theory (DFT) calculations showed that the high growth rate of WSe_2_ was caused by the small energy barriers for the diffusion of W and Se species on the Au substrate. Therefore, it can be concluded that surfaces with low active surface energy can facilitate the nucleation and growth of TMDCs at low temperature.

In addition, we can use additives to promote the delivery of precursors onto the substrate surface to accelerate the nucleation and growth of TMDC domains during CVD process. For example, Li et al*.* firstly reported the growth of WSe_2_ and WS_2_ monolayers at 700–850 °C by using the alkali metal halides (MX where *M* = Na or K and *X* = Cl, Br, or I) as the growth promoters. They found that these additives facilitated the formation of volatile tungsten oxyhalide species (WO_x_Cl_y_) at high temperatures, which improved the delivery efficiency of precursors onto the substrate surface (Fig. 9i). Due to the doping-free effect of alkali metal and halogen atoms, the FET based on WSe_2_ domain showed a high on/off current ratio of 10^7^ and a high hole mobility of 102 cm^2^ V^−1^ s^−1^ (Fig. 9j and k) [71]. On the basis of similar salt additive design, Zhou et al*.* prepared a wide range of 2D TMDCs, including 32 binary compounds based on the transition metals (Ti, Zr, Hf, V, Nb, Ta, Mo, W, Re, Pt, Pd, and Fe), 13 alloys (11 ternary, one quaternary, and one quinary), and two heterostructured compounds [72].

Moreover, CVD can realize the *in* situ doping of heteroatoms into TMDCs to tune their electronic structures with the introduction of additives and thus implement the conversion of *n*-type TMDC into *p*-type [73–75]. For example, Tang et al*.* reported the *in *situ doping of Nb into WS_2_. Bandgaps of monolayer WS_2_ were changed from 1.98 to 1.65 eV by varying the doping concentration of Nb from 0.3 to 4.7 at%. The Nb-doped WS_2_ had a high crystallinity, tunable compositions and property, as well as good uniformity. Electrical transport measurements showed that Nb-doping converted *n*-type WS_2_ into *p*-type. This change in the electronic properties and device characteristics is explained by DFT calculations, which demonstrated that the 4d electron orbitals of Nb dopant atoms contributed to the density of states around the Fermi level in Nb-doped WS_2_ and thus lowered the Fermi level into *p*-type region [76]. These works show that the additives provide a universal method to control the growth process of *p*-type 2D TMDCs and the electronic properties of 2D TMDCs.

Overall, the CVD method has been widely used to grow various 2D materials, but the thermodynamics and chemical reaction mechanisms are still difficult to understand. The researchers have not truly realized the controllable growth and mass production of 2D materials via CVD. In addition, the CVD process usually needs to be carried out at high temperatures, which hinders the utilization of some flexible and low melting point substrates (*e.g*., polyimide) for the construction of wearable and smart devices.

#### MOCVD

Compared with CVD, MOCVD uses metal–organic compounds as the reaction precursors. Usually, the liquid metal–organic precursors (metal precursors) and diethyl sulfide precursors (non-metal precursors) are introduced into the growth chamber by carrier gases to participate in the chemical reaction on the substrate surface. The flow rate of gaseous precursors can be precisely controlled during the growth process, and this feature directly contributes to the high controllability of MOCVD. So, MOCVD has been widely used in semiconductor manufactures.

**Fig. 10:**
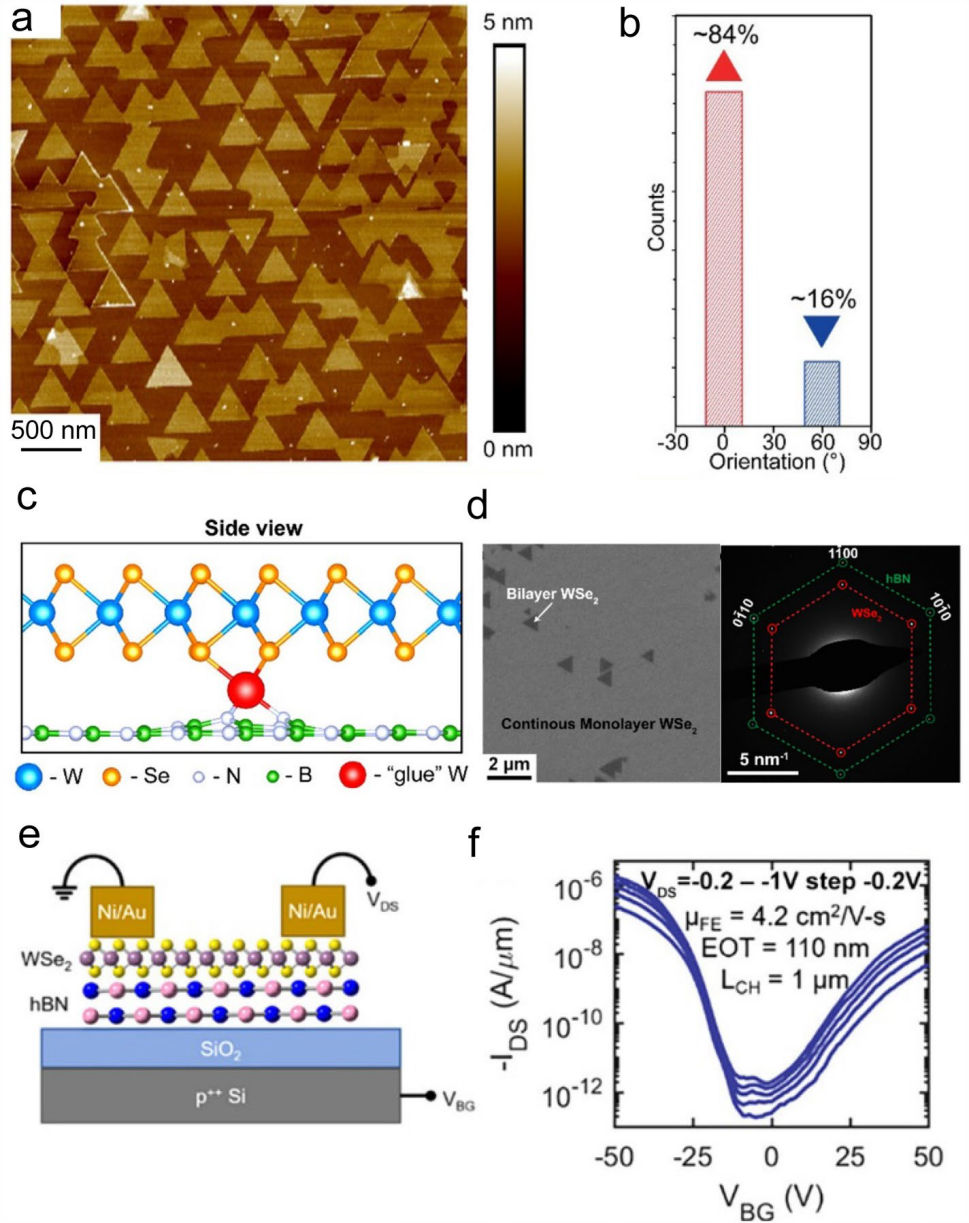
MOCVD growth of *p*-type 2D semiconductors. **a** AFM image of the epitaxial WSe_2_ domains grown on the h-BN substrate. **b** Orientation histogram of the 0°- and 180°-oriented WSe_2_ domains on the h-BN substrate. **c** The relaxed DFT structure of a W interstitial atom (red) sandwiched between a pristine WSe_2_ flake and a boron vacancy in h-BN. **d** SEM image of the monolayer WSe_2_ film on the h-BN substrate and the corresponding SAED pattern showing the epitaxial relationship between the WSe_2_ domain and the h-BN substrate. **e** Schematic of the back-gated WSe_2_ FET on 10-nm thick h-BN dielectric layer supported on 100-nm SiO_2_/p.^++^Si with Ni contacts. **f** *I*_d_-*V*_g_ curves at various *V*_d_ with a step of 0.2 V [82]

So far, great progress has been made in the MOCVD growth of large-area thin TMDC films (*e.g.*, MoS_2_, WS_2_, and WSe_2_) [77–81]. As for the preparation of *p*-type 2D materials, Zhang et al*.* used the gas source MOCVD reactor to grow monolayer WSe_2_ films at 800 °C with W(CO)_6_ and H_2_Se as precursors and H_2_ as the carrier gas. The as-grown 2D WSe_2_ flakes were well aligned on the exfoliated single-crystal h-BN substrate (Fig. 10a and b). First principles calculations showed the mechanism behind this high alignment: single-atom vacancies in h-BN trapped W atoms during the growth process (Fig. 10c) and thus facilitated the nucleation of WSe_2_ domains with the same orientation and the formation of the continuous thin film (Fig. 10d). In addition, the selected-area electron diffraction (SAED) pattern of the film confirmed the in-plane epitaxial relationship as < 1–100 > WSe_2_ ||< 1–100 > h-BN with 0° misorientation feature (Fig. 10d). The WSe_2_-based FET showed an ambipolar transport behavior and with a hole mobility of 4.2 cm^2^ V^−1^ s^−1^ (Fig. 10e and f) [82].

MOCVD has laid a solid foundation for the preparation of wafer-scale 2D TMDCs, which provides a platform for the next transfer and assembly process to prepare the heterojunctions. However, it should be emphasized that the growth rate of MOCVD is relatively slow, and the precursors of MOCVD are highly toxic. These two main drawbacks hinder the development of MOCVD in the preparation of 2D TMDCs.

#### Post-Treatment

Post-treatment has been made to tune the electronic structure of 2D materials, including charge transfer doping induced by the absorption of other molecules or functional groups [83–85] and plasma treatment [86–88]. However, these methods have some deficiencies. For instance, charge transfer doping is unstable as the device performance usually evolves with time, whereas plasma treatment may cause defects and damage the original structure of the 2D materials. So, the post-treatment technique of 2D materials should be optimized to realize the precise performance modification of 2D materials.

**Fig. 11:**
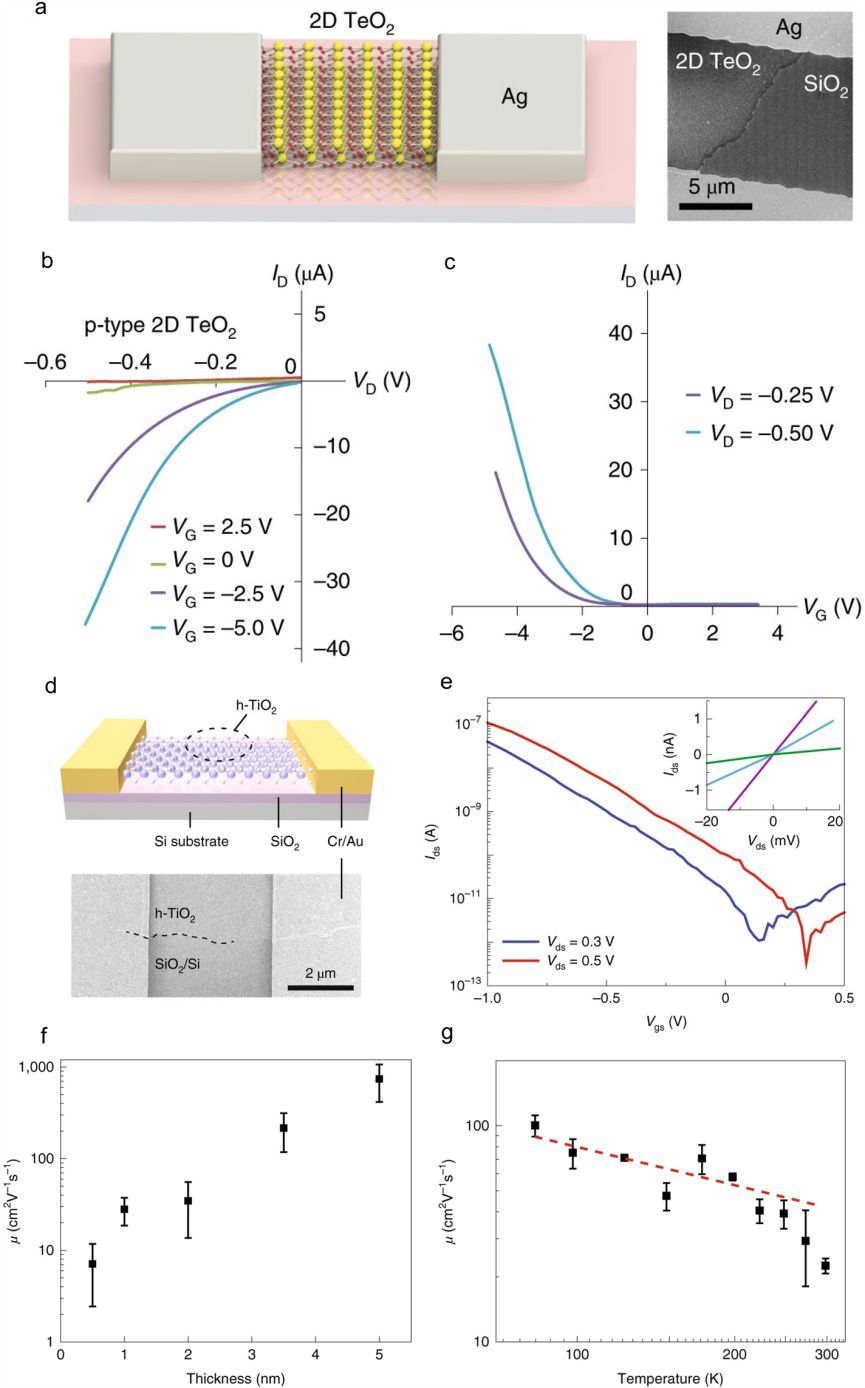
Post-treatment method to grow *p*-type 2D semiconductors. **a** Schematic and SEM image of 2D *β*-TeO_2_-FET. **b** Output and **c** transfer curves of the FET [35]. **d** Schematic and SEM image of 2D *h*-TiO_2_-based FET. **e** Transfer curves obtained from a 1-nm thick *h*-TiO_2_ device at room temperature, the inset is the output curves of the FET at *V*_g_ of −1 V (purple), −0.9 V (blue), and −0.8 V (green). **f** Field-effect mobility as a function of the thicknesses of 2D *h*-TiO_2._ **g** Field-effect mobility as a function of the temperature for the device shown in f, the red dashed line indicates the power-law dependence between mobility and temperature (*μ* ≈ *Τ*^−0.5^) [36]

One of the typical and effective post-treatment is oxidation process, and it can fulfill the controllable growth of novel 2D semiconducting oxides. For example, Zavabeti et al*.* reported a roll transfer method to grow bilayer *β*-TeO_2_. The tip of a glass rod with molten Te precursor swept across the substrate. Then, after an oxidation treatment with well-modified parameters, *e.g.*, oxygen dosage, growth time, droplet diameters, and deposition velocity, *β*-TeO_2_ nanosheets were formed on the substrate with tunable substrate coverage and lateral dimensions (Fig. 11a). FET based on the *β*-TeO_2_ nanosheets showed the *p*-type behavior with a high on/off current ratio of 10^6^ and high hole mobilities of 232 cm^2 ^V^−1^s^−1^ at room temperature and 6000 cm^2^ V^−1^ s^−1^ at 220 K (Fig. 11a-c) [35]. In another work, Zhang et al*.* reported the growth of monolayer and few-layered hexagonal TiO_2_ (*h*-TiO_2_) by strictly controlling the oxidation process of Ti at the metal–gas interface. They firstly polished the bulk Ti surface to minimize the roughness and suppress the defect-driven promotion of oxidation. An oxygen-deficient environment was provided to slow down the process of oxygen penetration into the Ti lattices. Then, uniform oxide layer was then grown at an optimum temperature. Finally, the formed TiO_2_ films were mechanically exfoliated and transferred onto various substrates for electronic applications. FET based on the *h*-TiO_2_ showed a *p*-type transport performance with a hole mobility of 950 cm^2^ V^−1^ s^−1^ at room temperature (Fig. 11d–g) [36]. Overall, this method is universal to grow post-transition metals, lanthanides, and metalloids in principle. More efforts to control the thickness of post-processing samples, which we can learn from the silicon industry to make this method more accurate and efficient.

## Applications of *p*-Type 2D Semiconductors

The discovery and successful preparation of high-quality *p*-type 2D semiconductors facilitate their application in electronic and optoelectronic devices. They can be integrated with *n*-type 2D semiconductors to construct high-performance electronic devices, and the unique electronic, magnetic, and optical properties of these *p*-type 2D materials can endow these cutting-edge devices with novel functionalities.

### *p–n* Junctions

**Fig. 12:**
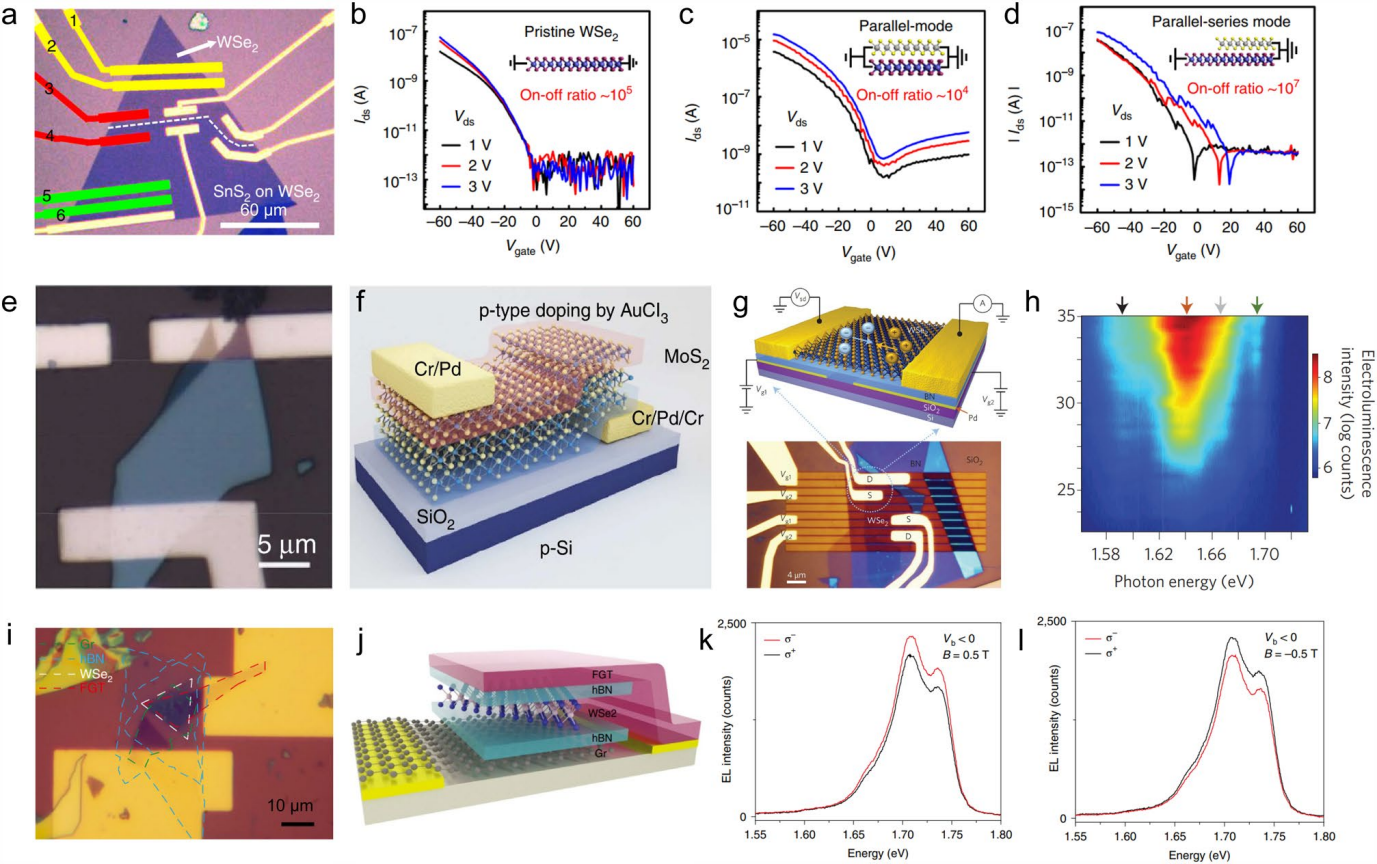
Electronic devices based on *p*-type 2D semiconductors and their heterostructures. **a** Multi-electrode FETs based on WSe_2_/SnS_2_ heterostructures, in which *p–n* junctions formed with different contact modes. **b-d** Output characteristics of the *p–n* junctions with different contact modes [89]. **e, f** OM image and schematic of the vertical MoS_2_-based *p–n* homojunctions [93]. **g** OM image and schematic of the WSe_2_ *p–n* homojunction devices for LED application. **h** EL intensity plot as a function of bias current and photon energy of the valley-LED [100]. **i, j** OM image and schematic of the WSe_2_-based valley-LED with FGT as the tunnel contact. **k, l** Polarization-resolved EL spectra for σ^−^-polarized and σ.^+^-polarized detection with the magnetic field pointed outwards and inwards toward the WSe_2_ surface [103]

Through manual stacking or direct growth, *p*–*n* junctions, one of the most basic electronic devices, can be constructed by vertically or horizontally integrating the *p*-type 2D domains with 1D, 2D, or 3D *n*-type materials. For instance, based on the CVD-grown vertical vdW WSe_2_/SnS_2_ heterostructures, Yang et al*.* fabricated large-scale bilayer *p–n* junctions with different contact modes on the SiO_2_/Si substrate [89]. As shown in Fig. 12a–d, multi-electrode FETs were integrated on a single heterostructure, and the formed *p–n* junctions with different contact modes exhibited different output characteristics. But they all possessed excellent electronic and optoelectronic performances, including the ultra-low off-state leakage current of 10^−14^ A, high on/off current ratio of 10^7^, and fast photoresponse time of 500 μs. When acting as a photovoltaic device, this WSe_2_/SnS_2_ *p*–*n* junction showed a high photoresponsvity of 108.7 mA W^−1^ and photodetectivity of 4.71 × 10^10^ Jones [89]. For another example, Jiao et al*.* fabricated the 2D BP/3D HgCdTe heterostructures by stacking BP on the bulk HgCdTe with special microstructure design [90]. The formed *p–n* junction possessed a type-III broken-gap band alignment and performed well as a photodiode to detect mid-wave infrared (MIR) irradiation. It showed a high peak blackbody detectivity of 7.93 × 10^10^ cm Hz^1/2^ W^−1^ and an average blackbody detectivity over 2.1 × 10^10^ cm Hz^1/2^ W^−1^ in MIR region. More importantly, the anisotropic crystal structure of BP endowed it with a strong intrinsic linear dichroism [91], making it sensitive to the polarized incident light [92]. The BP/HgCdTe *p*–*n* junction inherited this feature and showed strong polarization sensitivity, making it a favorable candidate for the next-generation infrared detector for multi-information acquisition [90].

Besides, *p–n* homojunctions were also constructed by post-doping engineering of the 2D materials. When chemically doped with AuCl_3_ and benzyl viologen, an ultrathin vertical *p–n* junction formed in the few-layer MoS_2_ (Fig. 12e and f) [93]. The ultimate thickness of this *p–n* homojunctions reached as low as 3 nm, and they showed a thickness-dependent rectification behavior. Compared with the heterojunctions, this *p–n* homojunctions minimized the carrier lost and maximized the carrier transport efficiency. Together with the high flexibility, this MoS_2_-based *p–n* homojunctions have significant utility in flexible electronic and optoelectronic applications [93]. Similar BP-based vertical *p–n* homojunctions were fabricated by ionic gel gating, forming an electric-double-layer transistor (EDLT) [94]. The perpendicular built-in electric field efficiently drove the photogenerated electrons and holes into surface or bulk layers, which greatly enhanced the linear dichroism photodetection ability of the device [94]. Then, by using the selected-area doping method, lateral BP *p–n* homojunctions were fabricated and used in photodetection [95, 96]. Benefitting from the enhanced photothermal–electric and photovoltaic effects, the lateral BP homojunction yielded an ultra-high polarization ratio of 288 at 1450 nm incident light, high responsivity and detectivity of 1.06 A W^−1^ and 1.27 × 10^11^ cm Hz^1/2^ W^−1^, respectively [96].

Recently, most of the reported works focused on the abrupt junctions with atomically sharp transitions at the interfaces. However, the compositionally graded *p–n* junctions have also shown their advantages in the wide-range modulation of the bandgap. For the graded WS_2_/WSe_2_ junction, the light-emission energy can be varied from 2.1 to 1.7 eV, and the recombination zone can be tuned laterally in the *p–n* junctions. Benefiting from these superiorities, a continuous and reversible color-tunable light-emitting device can be fabricated based on this graded WS_2_/WSe_2_ junction [97]. So, graded junctions will be another developing direction in the area of 2D *p–n* junctions. Besides, it should be noted that the contact metal electrodes and their integration methods can affect the transport behavior of 2D semiconductors [98, 99]. The electrode engineering should be another concern in this area.

### LED

LED is one of the main utilities of 2D *p–n* junctions, and it can be integrated into silicon and silicon-on-insulator platforms. LED based on 2D *p–n* junctions not only shows excellent electroluminescent performance but also has some novel features owing to the unique electronic, optical, and magnetic performances of 2D materials. For example, the LED based on the lateral WSe_2_ *p–n* junctions yielded bright electroluminescence (EL) with a small injection current, and the EL was tuned between regimes of impurity-bound, charged and neutral excitons by changing the injection bias (Fig. 12g and h) [100]. Interestingly, for the EL that comes from the valley excitons formed at the ± K valleys where valley coherence of excitons is generated [100–102], spin- and valley-LEDs with controllably polarized emission were realized owing to the spin-valley locking effect when using ferromagnetic contacts to inject spin-polarized charge carriers into the valleys [100]. As reported by Li et al*.*, valley polarization was successfully generated by using ferromagnetic metal Fe_3_GeTe_2_ (FGT) as the tunnel contact to inject spin-polarized holes into monolayer WSe_2_ (Fig. 12i and j) [103]. The output EL became polarized, and the helicity of EL flipped its sign by changing the external magnetic field direction (Fig. 12k and l) [103]. Moreover, the WSe_2_-based LEDs were integrated with active photonic nanostructures, *e.g*., waveguide and microcavity, toward the real application in optoelectronic chips with reduced footprint and higher integration capacity owing to the atomically thin and planar nature of WSe_2_ [104]. When integrated with CdS nanoribbon, the WSe_2_ LED fully leveraged the CdS waveguide to realize efficient optical routing of EL emission, which made this LED possible to interconnect with other optoelectronic units (*e.g*., modulators and photodetectors) in the photonic-integrated circuits [104].

MIR light emitting has a wide range of applications in optical communication, thermal imaging, medical treatment, and material analysis applications. However, it is beyond the limit of traditional Ge- and Si-based light-emitting devices [105]. The bandgap of BP ranges from 0.3 eV (bulk) to 2 eV (monolayer), and the bandgap can be controlled below 0.441 eV for the BP with 8 or more layers. This well-modified bandgap allows the BP-based LED (BP-LED) to emit bright MIR light [106]. Chen et al*.* reported that the wavelength of the BP-LED emitted MIR light was widely modulated by the external electrical field [107]. The photoluminescence (PL) peak of a ~ 20-layer BP flake was continuously tuned from 3.7 to 7.7 μm by increasing the field intensity [107]. Besides, the wavelength of EL emitted from BP was reversibly tuned via strain engineering by taking the advantage of strain-sensitive bandgap of BP [108]. More importantly, the emitted MIR light from BP-LED was polarized due to the anisotropic crystal structure of BP [109]. The EL intensity reached the maximum along the armchair axis and the minimum along the zigzag axis of BP. The intensity ratio along the two directions exceeded 7 [110]. However, the efficiency of these MIR BP-LEDs is still low at present, *i.e*., ~ 1% internal efficiency and ~ 0.03% external efficiency for the BP/MoS_2_ heterojunctions [109]. Many efforts have been made to solve this problem. For instance, by integrating the BP-LED with an Al_2_O_3_/Au optical cavity, the external efficiency increased to 4.43% [111]. The wall-plug efficiency of this resonant-cavity LED also reached 1.78% with a maximum optical power density of 2.17 W cm^−2^ [111].

Despite the great achievements mentioned above, there are still many issues that remained to be solved or figured out in LEDs based on the 2D *p–n* junctions, including the luminescence mechanism and fabrication of large-scale devices. To be specific, more efforts should be exerted to further understand the charge transfer processes, exciton spin and valley relaxation dynamics in these 2D LEDs. Further efforts are also needed to improve the luminescence efficiency because the external quantum efficiency (EQE) of these 2D LEDs is still lower than the traditional LEDs based on III–V semiconductors [112]. Besides, the 2D LEDs mentioned above are all based on the CVD-grown or micromechanically exfoliated and stacked *p–n* junctions. Limited by the size of the raw material, fabrication of large-scale and reliable 2D LEDs can be hardly fulfilled, which will become one of the biggest obstacles to making these 2D LEDs meet the requirements of practical applications.

### Photodetector and Other Photonic Devices

Except for LED, these *p*-type 2D materials and their heterostructures can also be used to fabricate other photonic devices such as photodetector, optical modulator, laser source, quantum emission, and nonlinear optical devices [113–115]. These devices exhibited marvelous characteristics. For example, the photodetector based on *p*-type vanadium-doped MoSe_2_ with a certain degree of Mo vacancies displayed a broadband spectral response from 365 nm (ultraviolet) to 2240 nm (infrared), and it had high responsivities of 9700 and 2800 mA W^−1^ at 520 and 2240 nm, respectively [116]. In another work, the optical modulator based on BP passively modulated broadband lasers with the wavelength ranging from 639 nm (red) to 2.1 μm (MIR) [117]. Interestingly, site-controlled single-phonon quantum emitters were created through strain and defect engineering based on WSe_2_ flakes. The emitters worked at a relatively high temperature at 150 K and showed a single-photon purity of > 95% [118].

All the aforementioned photonic devices based on *p*-type 2D materials can be integrated together or with other 2D devices on conventional silicon photonic platforms [115, 119, 120]. Many researchers have attempted to manufacture photonic circuits based on 2D materials. BP-based LED, photodetector, and modulator, as well as WSe_2_-based single-photon emitter have already been integrated into the photonic circuits [121–125], and these attempts have tentatively explored the feasible method to fully integrate 2D nanophotonic and quantum photonic circuits.

### Complementary Inverters

**Fig. 13:**
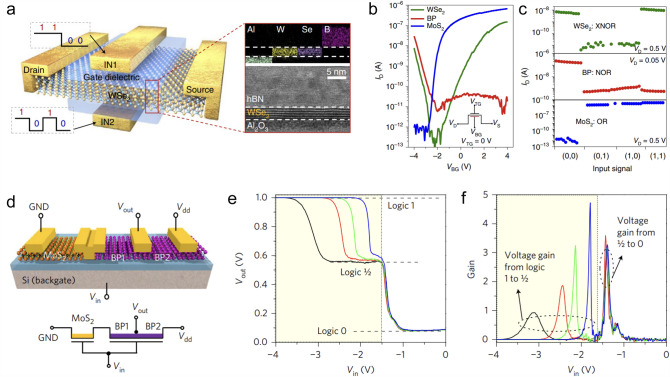
Application of *p*-type 2D semiconductor in CMOS inverters. **a** Schematic of the WSe_2_-based inverter. **b** Transfer curves of the inverters based on WSe_2_ (ambipolar type), BP (p type), and MoS_2_ (n type). **c** Logic behaviors of XNOR, NOR, and OR realized in the inverters [132]. **d** Schematic of the ternary inverter based on MoS_2_/BP FETs. **e** Plot of V_out_ versus V_in_ and **f** voltage gain of the ternary inverter, which clearly shows the three logic states, i.e., logic 1, 1/2, and 0 [141]

Complementary inverters are the fundamental units in logic circuits. Both the *p*-type and *n*-type transistors are the essential components of complementary metal oxide semiconductor (CMOS) inverters [126]. So, with the *p*-type BP, WSe_2_, b-AsP, metal oxide, and other doped TMDCs, 2D complementary CMOS inverters were constructed. For example, based on the CVD-grown lateral WS_2_-WSe_2_ heterostructures, Duan et al*.* fabricated the complementary inverters with a high-voltage gain of 24 [127]. Even in a single WSe_2_ flake, complementary inverters were implemented by selected-area surface charge transfer doping to fabricate both *n*-type and *p*-type FETs at the same time or through a doping-free contact engineering strategy [98, 128, 129]. The WSe_2_-based complementary inverter not only exhibited excellent performance, *e.g*., long retention time (> 500 s), low operating source-drain current at the order of nanoampere, and the hysteresis window located at 0 V [130], but also had complex logic functions. AND, NAND, NOR, XOR, and stable multi-valued logical states were all realized in the inverter with WSe_2_ as the channel material (Fig. 13a–c) [98, 131–133]. Similarly, researchers also have fabricated complementary inverters based on BP and its heterostructures. A high gain value of 33 was achieved in the inverter fabricated on a single BP flake with spatially-controlled aluminum doping to establish the *p–n* homojunctions [134], and multi-valued logic states were observed in the BP heterostructures such as BP/SnSe and BP/MoS_2_ [135, 136].

With the improving controllable doping technique, complementary inverters were manufactured by integrating the substitutionally doped *p*-type and undoped *n*-type TMDC flakes together or through the localized doping strategy to form *p–n* homojunctions. For example, by integrating the *p*-type FET based on the substitutionally doped WS_0.3_Se_1.7_ with the *n*-type WS_2_ FET, Kuddus et al*.* obtained an electrically isolated complementary inverter with a gain of 4–5 [137]. In another work, with the help of photoresist shielding layer, localized oxygen plasma treatment was performed on the selected MoS_2_ flake or just the partial area of a single MoS_2_ flake to form a *p*-type channel in the* n*-type matrix, and then, high-performance complementary inverters were directly made on this homojuntion [138, 139]. With the laser irradiation treatment, *α*-MoTe_2_ was selectively converted to form *p*-type regions because of the oxygen doping effect, and inverter was realized on the single MoTe_2_ domain with the formed *p–n* homojunction [140].

These 2D complementary inverters can incorporate other functionalities, including memory, amplifier, and photodetection, etc. The BP/ReS_2_ heterostructures not only modulated the logic states of “1,” “1/2,” and “0” to act as a nonvolatile ternary logic inverter but also mimicked the trilingual synaptic response with a high synaptic weight change over 10^4^% (Fig. 13d–f) [141]. So, a logic-in-memory device was realized, and this synaptic device arrays fulfilled the artificial neural network simulation for handwritten digits recognition with an accuracy of around 90% [141]. The ambipolar transport feature of hBN-encapsulated BP-based FET allowed it to operate as a type-switchable logic inverter with a voltage gain of 6 and an operating frequency of up to 160 kHz. Meanwhile, this BP-based FET also served as an inverting or noninverting amplifier controlled by the polarity of the input and supply voltage [142]. In another work, by using MoS_2_ and WSe_2_ as the *n*-type and *p*-type channels on glass substrate, the formed inverter showed superior performances, *e.g*., a maximum voltage gain of 27, sub-nanowatt power consumption, and almost ideal noise margin [143]. This device realized NOT, OR, and AND logic functions and operated as a push–pull circuit for organic LED (OLED) pixel switch. Similar results were achieved on BP inverters fabricated on glass substrate [144]. Further, three fundamental functionalities, including photodetector, inverter, and alternating current (AC) rectifier, were integrated in one *p*-WSe_2_/*n*-InGaZnO device [145]. These above-mentioned achievements have laid a solid foundation for the sense-memory-computational integration. However, the present material synthesis and device fabrication techniques can hardly guarantee the high uniformity of the material and device properties. Before solving this problem, such complicated and multi-functional devices cannot be fulfilled based on these 2D semiconductors with robust performance.

### System Integration

**Fig. 14:**
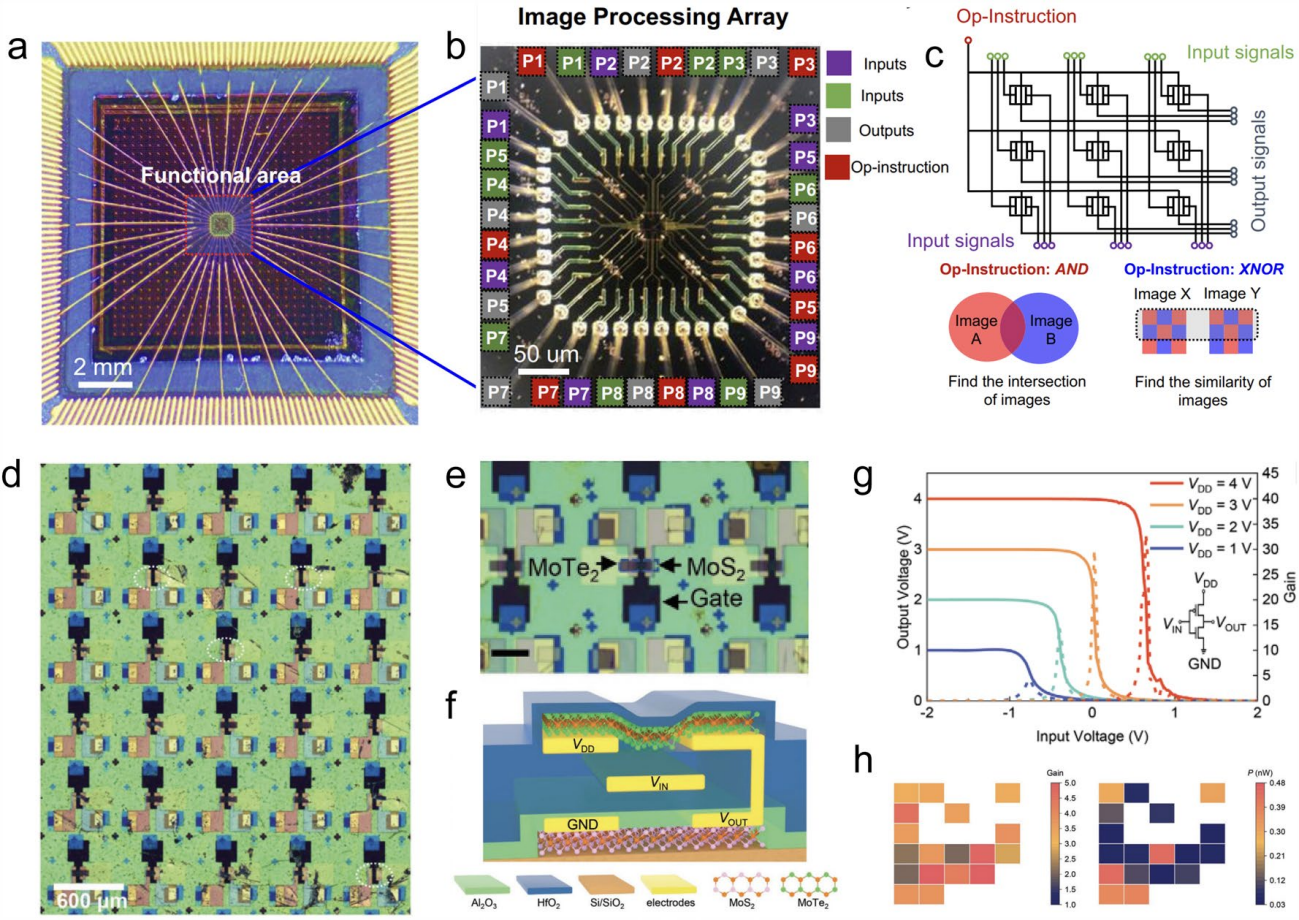
System integration based on *p*-type 2D devices. OM image of the on-chip system for **a** image processing and **b** the 3 × 3 processing array. **c** Schematic circuit diagram of the pixel processing array [147]. **d** Large-scale monolithic 3D architecture CMOS inverter array. **e** Magnified OM image and **f** structure of the inverters. **g** Voltage transfer characteristic and voltage gain plots of the inverter at various *V*_*D*_. **h** Statistic voltage gains and power consumptions of CMOS inverters in the device array [148]

With the improving preparation, transfer, and device fabrication techniques of 2D materials, efforts have been made in the system integration of 2D device arrays with specific functionalities. At present, 2D materials have been incorporated into silicon platforms for monolithic “on-silicon” or “with -silicon” circuits, in which silicon serves as the substrate or functional component, respectively [146]. So, the developing schedule of 2D system integration can be accelerated by drawing experience from the semiconductor industry. For example, to efficiently handle different graphics tasks, a 3 × 3 pixel processing array was fabricated based on a high-quality 90 × 60 μm^2^ WSe_2_ flake (Fig. 14a–c). Each pixel processing unit was realized by using a single WSe_2_ transistor as the logic function, in which AND and XNOR could be switched. Image intersection and comparison tasks were accomplished based on this processing arrays, and meanwhile, the energy consumption was reduced by 16% when compared to the traditional logic circuits [147]. Also, a large-scale CMOS inverter array was fabricated based on CVD-grown *n*-type MoS_2_ and *p*-type MoTe_2_ with a high device yield of about 60% (Fig. 14d–h) [148].

Except for the planar arrays, system integration of 2D devices has gone into the stage of monolithic 3D architecture. For example, Sivan et al*.* integrated the WSe_2_-based TFT and resistive random access memory to form the 1 transistor–1 resistor (1T1R) memory cells. Then by vertically stacking these TFT channels, 1T1R memory array with a high density of sub-0.01 μm^2^ was constructed, and this memory array is applicable for future high-density monolithic 3D memory systems [149]. A 3D XNOR logic array based on WSe_2_ transistors was made by Chen et al*.* to form a binary convolutional neural network. This novel architecture provided a computation density of 52.9 TOPS mm^−2^, power consumption of 7.3 mW, and energy efficiency of 622.35 TOPSW^−1^, and these performance parameters were all superior to the silicon-based and memristor-based systems [132].

To accomplish the complicated task of machine vision, Mennel et al*.* combined the functionalities of logic computing and photodetection together in the WSe_2_-based devices and fabricated an artificial neural network photodiode array [150]. This system successfully performed the real-time multiplication of the projected image with the photoresponsivity matrix and meanwhile adjusted and stored the synaptic weights in the neural network locally. After supervised and unsupervised learning and training, this system classified and encoded the inputted images with an extremely high throughput of 20 million bins per second without energy consumption [150]. This work encourages the future exploration of ultrafast machine vision based on the system integrations based on 2D *p*-type materials. But it should be noted that both the scale and processing technologies of these integrated 2D systems fabricated in these researchers’ laboratory lag behind the silicon-based very large-scale integration circuit (VLSI). Further breakthroughs in the material synthesis and device fabrication techniques are required to take these 2D system integrations into the consideration in the semiconductor industry but are not limited to academic researchers.

## Conclusions and Outlook

**Fig. 15:**
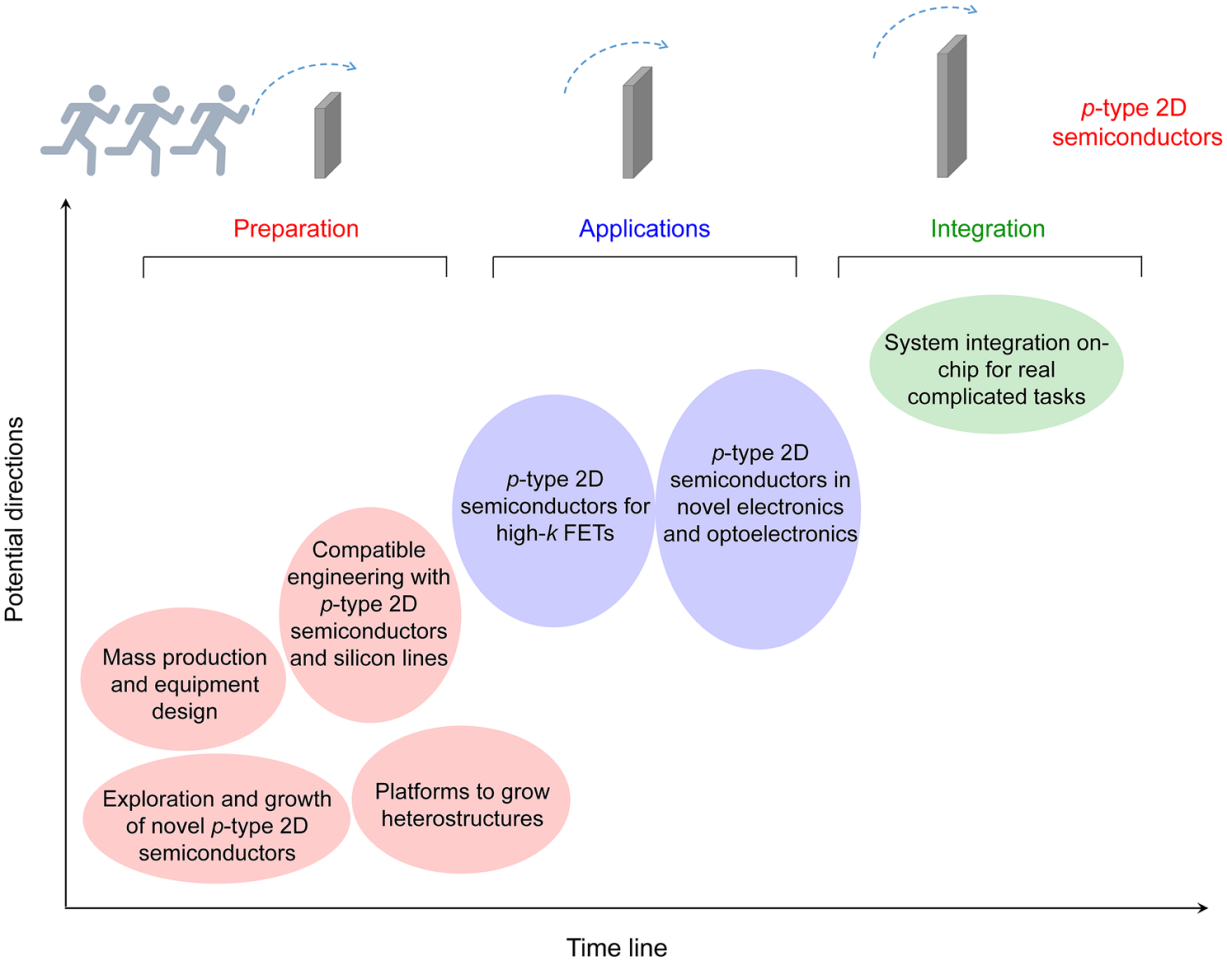
Roadmap of the research about *p*-type 2D semiconductors

In this review, we have summarized the state-of-the-art of *p-*type 2D materials. We reviewed the current attends on the preparation of various *p*-type 2D materials, including mechanical exfoliation, liquid-phase exfoliation, electrochemical exfoliation, MBE, PVT, PLD, CVT, CVD, and MOCVD methods. Then, we summarized the mainstream applications on the *p*-type 2D materials such as *p–n* junctions, LEDs, COMS inverters, and system integration. Despite its rapid progress, the development of *p*-type 2D materials is still in its early stages. A large number of other* p*-type 2D materials and their applications have not been well studied or have not been successfully prepared, and more interesting studies are waiting to be found. In the following text, we propose a few potential research directions in this emerging field (Fig. 15).

### Exploration and Growth of Novel p-Type 2D Materials

With the assistance of high-throughput computation based on the materials database, it is facile to find more *p*-type 2D materials with unusual structures and properties [151]. Here, we think that the following directions may be important and promising. (i) *p*-type 2D materials with high air stability. Despite that contact engineering, chemical doping, and electrostatic doping methods can be used to convert *n*-type devices to *p*-type ones, the direct synthesis of high-quality *p*-type 2D semiconductors and fabrication of *p*-type devices are also highly desirable and important. (ii) *p*-type 2D materials with high carrier mobility. High carrier mobility is always one of the most key factors to promote the performance of electronic and optoelectronic devices based on *p*-type 2D materials. (iii) Natural *p*-type layered materials which are abundant in earth.

### Using p-Type 2D Materials as Platforms to Grow Heterostructures

The large family of heterostructures may be grown by using *p*-type 2D materials as platforms. For example, by exposing the existed 2D materials (*e.g*., WSe_2_) to CVD setups with plasma or rapid thermal annealing, novel structures might be formed, such as metal single atoms [152], nanopores [153], and *Janus* structures [154]. In addition, the post-treatment of as-grown samples may also form new structures. Such treatments include carbonation [155, 156], nitridation [157], oxidation [158–160], phosphating [161], n-butyl lithium treatment [162, 163], as well as ion intercalation [164, 165].

### Mass Production and Equipment Design for the Growth of p-Type 2D Materials

With the assistance of gaseous organic metal compound precursors [77–79], one can improve the controllability of the concentration distribution, which is hardly controlled with solid precursors, facilitating the preparation of 2D materials with large scale. In addition, designing automatic programming equipment [166] will also help to promote the development of *p*-type TMDCs with mass production.

### Exploration of the Compatible Engineering of p-Type 2D Materials with the Silicon Production Line

We need to learn from the Si production line and make the *p*-type 2D materials compatible [167]. However, at the moment, the integration of synthetic 2D materials on SiO_2_/Si substrate is lacking production-suitable tools and associated infrastructure, which is holding back reproducible device fabrication and limits the high performance. Only a concerted effort to the fundamental research communities and the semiconductor industries can make them complement each other.

### Exploration of the High-k Dielectric Materials on p-Type 2D Semiconductors-Based FET Devices

There are several works reported that utilization the ultrathin high-*k* dielectric materials (*e.g*., BaTiO_3_, SrTiO_3_, HfO_2_, Sb_2_O_3_, and AlScN) as the thin equivalent oxide thickness (EOT) on 2D MoS_2_-based FET device, which show a high on/off current ratio of 10^6^ at an ultra-low operating voltage and the subthreshold swing approaches the thermionic limit of 60 mV dec^−1^ [168–173]. These high-*k* dielectric materials can also be used for the *p*-type 2D semiconductors-based FET devices in principle. Recently, the *in* situ conversion from semiconducting B_i2_O_2_Se to high-*k* dielectric Bi_2_SeO_5_ opens a new avenue to prepare native oxide layer on 2D materials [159, 174, 175]. In the future, integrating the native oxide with *p*-type 2D semiconductor will accelerate their use in electronics.

### Application of p-Type 2D Materials and their Heterostructures in Novel Design of the Electronic and Optoelectronic Devices

The unique physical properties of 2D materials will prompt the exploration of novel devices based on these *p*-type 2D materials. Yun et al*.* observed the long-range order in semiconducting vanadium-doped WSe_2_ at room temperature, and more interestingly, the ferromagnetic order can be modulated by the gate voltage [176]. This superior feature makes the *p*-type WSe_2_ a popular channel material in the future spintronic devices. For another example, Jiang et al*.* fabricated the ferroelectric FETs (FeFETs) based on WSe_2_/CuInP_2_S_6_ heterostructures with a buried-gated design, which showed the clear clockwise hysteresis loop with an on/off current ratio of 10^5^, endurance cycles of more than 100, and retention time of 50 s [177]. The FeFET design facilitated the quasi-nonvolatile memory devices and promoted the data storage with a low-power consumption [178–181]. Besides, these atomically thin materials also facilitated the incorporation of multifunctionalities in one single device. For example, the self-propagated defects generated unique optoelectronic functionalities in BP [182]. The BP-based proof-of-concept device not only distinguished the radiations between UV-A (365 nm) and UV-B (280 nm) but also showed a light-stimulated synaptic response for neuromorphic computing, and then was used to perform digital logic operations using light.

### System Integration On-Chip or With-Chip for Complicated Tasks

Wafer-scale device fabrication of *p*-type 2D materials is another important developing direction to realize the system integration for complicated tasks, *e.g*., machine vision and machine learning. Now, large-scale 2D devices have been integrated on the conventional silicon substrate following an on-silicon or with-silicon strategy. Most of the aforementioned works follow the first strategy, in which the conventional silicon substrate is just used to support the 2D devices and the integrated system. For the with-silicon strategy, 2D device systems can be organically combined with the silicon-based functional devices to exert the utmost advantage of the novel 2D and current 3D systems [183]. Wang et al*.* have summarized several works and gave a prospect of the incorporation of 2D and 3D architectures, showing the prototype monolithic with-silicon hybrid integrated circuits for enhanced performance and enriched functionality [146]. However, further efforts, involving the material preparation, device fabrication, and chip packaging, are needed to accomplish the large-scale integration and make them steadily operate for real tasks.

In conclusion, although many related studies are still underway, we anticipate exciting achievements in both fundamental research and applications of *p*-type 2D materials. We hope that this review can appeal to researchers to devote unflagging efforts to the exploration of 2D materials in the future.
